# Unlocking precision diagnostics: A multimodal framework integrating metabolomics with advanced machine learning techniques

**DOI:** 10.1371/journal.pone.0318473

**Published:** 2026-06-15

**Authors:** Parisa Shahnazari, Kaveh Kavousi, Hamid Reza Khorram Khorshid, Bahram Goliaei, Reza M. Salek

**Affiliations:** 1 Laboratory of Complex Biological Systems and Bioinformatics (CBB), Department of Bioinformatics, Institute of Biochemistry and Biophysics (IBB), University of Tehran, Tehran, Iran; 2 Department of Bioinformatics, Kish International Campus, University of Tehran, Kish, Iran; 3 Genetics Research Center, University of Social Welfare and Rehabilitation Sciences, Tehran, Iran; 4 Personalized Medicine and Geno-metabolics Research Center, Hope Generation Foundation, Tehran, Iran; 5 Laboratory of Biophysics and Molecular Biology, Institute of Biochemistry and Biophysics (IBB), University of Tehran, Iran; 6 School of Clinical Medicine, University of Cambridge, Cambridge Biomedical Campus, Cambridge, England; Korea University - Seoul Campus: Korea University, KOREA, REPUBLIC OF

## Abstract

Integrating multiple omics modalities is a crucial strategy in cancer research, particularly in metabolomics, enabling early detection and detailed exploration of cancer biomarker signatures. This study evaluates five strategies for integrating metabolomics data from liquid chromatography-mass spectrometry, gas chromatography-mass spectrometry, and nuclear magnetic resonance. Deep Transfer Learning and Multiple Kernel Learning demonstrated superior performance, significantly improving classification accuracy, sensitivity, and robustness compared to single-modality analyses. Deep Transfer Learning employed a custom autoencoder for feature extraction followed by artificial neural network classification, while Multiple Kernel Learning optimized kernel matrices across different modalities. Feature extraction in the Deep Transfer Learning approach, combined with the selection of important features and subsequent analysis, revealed elevated levels of monounsaturated phospholipids such as phosphatidylcholine 30:1, phosphatidylethanolamine 32:1, and sphingomyelin 32:1 in HER2-positive cases. Additionally, β-alanine, gluconic acid, and N-acetylaspartic acid were increased, whereas 5’-deoxy-5’-methylthioadenosine and nicotinamide were decreased. These methods advance cancer detection, biomarker discovery, and the development of precise diagnostic and therapeutic tools while offering robust and adaptable strategies for multi-omics data integration across diverse biological datasets.

## 1. Introduction

Metabolomics, the comprehensive study of small molecules within biological systems, holds immense potential for elucidating the intricate molecular metabolic profiles associated with human diseases [1]. However, comprehensively detecting all metabolites using a single analytical technology presents significant challenges due to their diverse chemical nature and the limitations of current analytical instruments. As a result, a typical metabolomics experiment using a single analytical technique can only provide a partial identification or discovery of the full spectrum of metabolites within a system. Common analytical platforms employed in metabolomic investigations include liquid chromatography-mass spectrometry (LC-MS), gas chromatography-mass spectrometry (GC-MS), and nuclear magnetic resonance spectroscopy (NMR) [2]. Holistic coverage of the metabolome can be achieved by integrating NMR, GC-MS, and LC-MS for comprehensive analysis. Each platform captures a unique set of metabolites, collectively offering a more detailed perspective [2,3]. NMR detects high-concentration compounds with limited sensitivity; GC-MS is ideally suited for volatile compounds; and LC-MS is versatile, covering a broad spectrum of compounds and concentrations with high sensitivity [4]. *Fatema Bhinderwala* and colleagues (2018) highlighted the benefits of combining mass spectrometry and NMR for improved detection and annotation [5].

Multi-modal integration, or multi-view, multi-platform, or cross-modal integration, is a powerful strategy that significantly reduces false positives and negatives [6]. By implementing integration techniques, analytical confidence is notably bolstered as results are validated across platforms, thus enhancing the reliability and robustness of the data [7]. Moreover, integrating different platforms amplifies the likelihood of discovering previously unidentified metabolites through cross-referencing various spectra or analytes, effectively resolving ambiguities in compound identification [1]. In addition, comprehensive coverage in metabolomic studies contributes to a more profound understanding of metabolic pathways, enabling a more detailed pathway analysis [6]. Integrating multimodal metabolomics data is a valuable strategy in cancer research. It helps identify early detection biomarkers and monitor perturbations in metabolic profiles in biological fluids and tissues before clinical symptoms manifest. This proactive approach facilitates early disease diagnosis and is crucial in predicting clinical outcomes, advancing personalized medicine, and drug discovery [1,8,9].

On the other hand, integrating multiple platforms can be complex and challenging. This process requires careful consideration due to resource-intensive aspects that involve intricate sample preparation procedures, substantial time commitments, and the need for specialized expertise. Addressing these challenges is essential to ensure reproducibility, facilitate a smooth integration process, and derive meaningful interpretations from the diverse analytical techniques. Integration methodologies can be broadly categorized as either graph-based or non-graph-based, depending on the characteristics of the datasets and the functionalities of the techniques employed. *Eugene Lin* et al. (2017) investigated the integration of multimodal data in omics by applying machine learning and systems genomics approaches [10]. *Milan Picard* et al. (2021) categorized five integration strategies: early, mixed, intermediate, late, and hierarchical. This classification also involved categorizing methods by mathematical approach, such as network-based, deep learning-based, kernel-based, and matrix factorization-based approaches for integrating multi-omics data [8]. *Yanlin Wang* et al. (2022) have further ascribed the concept of intermediate integration by categorizing it based on matrix factorization, component analysis, multiple kernel learning, similarity network, Bayesian Network, Graph Transformation, and Artificial Neural Networks [11]. Nasim Vahabi *et al.* (2022) categorized unsupervised multi-omics data integration into three comprehensive categories: Regression/Association-based, Clustering, and Network-based Methods [12]. Additionally, *Francis E. Agamah* (2022) investigated network-based integrative multiomics [13]. Furthermore, *Daniyal Rajput* et al. (2023) observed stabilization in both effect sizes and ML accuracies beyond a certain sample threshold [14]. This finding suggests that even a modest sample size can suffice for analysis when dealing with high-quality datasets. The study proposed criteria for determining the appropriate sample size, emphasizing the importance of substantial effect sizes (≥ 0.5) and a high machine-learning accuracy threshold (≥ 80%). This recommendation is particularly valuable for metabolomics data, where sample numbers are typically limited.

This study evaluates five integration methodologies for multimodal data to identify the most effective approaches in metabolomics. The methods include straightforward concatenation, concatenated-ensemble, deep forest, multiple kernel learning, and deep transfer learning. In the deep transfer learning approach, features extracted from the encoder are weighted, and key features are selected for further analysis to identify significant biomarker signatures.

The primary objective of this research is to develop a robust pipeline applicable to medicine, enhancing the precision and depth of insights provided by analytical models. The integration techniques employed in this study can be summarized as follows:

### Straightforward concatenation

Straightforward concatenation is a common method for horizontally combining datasets with identical samples and class labels, frequently used in biological research to achieve more comprehensive insights. By expanding the dataset’s feature space, this approach enhances pattern recognition by integrating diverse platform data. However, it also presents challenges such as increased dimensionality, potential redundancy, platform compatibility assumptions, and data quality and interpretability concerns. While feature selection can help reduce dimensionality and redundancy, it cannot fully address platform compatibility and data quality issues. Additional preprocessing steps, such as normalization or alignment of datasets, are often required to ensure compatibility. Furthermore, this fusion technique typically serves as an initial step for integrating advanced machine learning methods, further enhancing the dataset’s analytical power and interpretability [15–17].

### Concatenation-Ensemble

The Concatenation-Ensemble method employs ensemble learning, which integrates multiple learning algorithms to enhance predictive performance beyond that of individual models. This approach involves the horizontal integration of datasets from diverse sources, followed by applying ensemble techniques such as Stacking, Bagging, AdaBoost, or Voting on the consolidated dataset. To ensure the robustness and reliability of the model, cross-validation is employed, validating its performance across various data subsets. By leveraging the strengths of multiple models and compensating for their weaknesses, this strategy significantly improves overall predictive accuracy and generalization capabilities [15].

### Deep forest

Deep Forest is an advanced ensemble learning technique introduced by Zhou and Feng in 2017, designed for both classification and regression tasks [18]. It leverages multiple layers of tree-based models, typically random forests, in a cascading architecture where each layer processes the input data and the output from the previous layer, including class probabilities or regression estimates. This hierarchical structure allows the model to represent features at different levels of detail, effectively capturing complex data patterns. The process starts with an initial layer that applies tree-based models to the original input features. Each subsequent layer then receives a combination of the original features and the outputs from the previous layer, refining the data representation and enhancing predictive accuracy [19,20].

### Multiple Kernel Learning

Multiple Kernel Learning (MKL) is a robust paradigm that addresses diverse learning challenges, particularly in classification and regression. Jérôme Mariette et al. (2017) and Xingheng Yu *et al*. (2020) underscored MKL’s efficacy when applied to breast cancer heterogeneous data, demonstrating commendable performance outcomes [21,22]. The essence of MKL lies in its ability to seamlessly integrate information from multiple sources, as reflected in the combined kernel function k (x, x’):


$$\begin{array}{c} \text{k}\left(\text{x},{\text{x}}^{\prime }\right)=\sum_{m=\text{1}}^{M}\beta_{\text{m}}\;k_{m}(x,x^{\prime }) \end{array}$$

where

k (x, x’) characterizes the combined kernel function between two input data points, x and x,’ considering information from M distinct platforms.

The summation $\sum_{m=\text{1}}^{M}\;$ denotes the weighted sum of individual kernel functions.

km (x, x’) denotes the individual kernel function associated with the m-th platform.

$\beta_{m}$ represents the weight or coefficient assigned to the m-th individual kernel [23].

The weights $\beta_{m}$ play a crucial role in adjusting the influence of each platform on the combined kernel.

### Deep transfer learning

Transfer learning is an advanced machine learning technique that employs knowledge gained from one task or dataset to enhance performance on a related task or a different dataset. Unlike traditional models trained independently for each task, transfer learning draws on insights from earlier tasks to improve generalization and adaptability in new situations. This method is particularly beneficial in scenarios with limited data, allowing for more effective learning by utilizing established features and patterns [24].

Conventional transfer learning modifies models for related tasks by integrating knowledge from one domain to improve performance in another. In contrast, concatenation transfer learning combines features from multiple sources before feeding them into the model, providing a more comprehensive understanding when each source contributes unique information [25].

This study applies transfer learning using a custom autoencoder architecture consisting of an encoder and a bottleneck (hidden layer). The encoder compresses input data into a lower-dimensional representation, capturing the most essential features through the bottleneck. However, unlike traditional autoencoders, the decoder is not used for data reconstruction. Instead, the bottleneck layer serves as the key feature extraction point, condensing data into a compact, informative form. In addition, a pre-trained encoder, previously trained on a related task, is employed to capture general patterns and feature representations. This pre-training provides a strong initialization, enabling the transfer of learned features to the new task, and improving performance, especially on limited datasets, by reducing the need for extensive re-training and speeding up convergence [26,27].

### SHapley Additive exPlanations (SHAP)

SHAP (SHapley Additive exPlanations) is a unified framework for interpreting machine learning model predictions based on game theory’s Shapley values [28]. It calculates each feature’s contribution to a prediction by comparing predictions with and without that feature across different combinations of other features. SHAP helps identify important features by assigning importance values representing their average marginal contribution to model predictions [29,30]. Key advantages include its strong theoretical foundation with properties like local accuracy and consistency, its ability to work with any machine learning model (model-agnostic), and its provision of global and local feature importance interpretations. In cancer research, SHAP helps identify key biomarkers influencing tumor development and treatment response, enabling more precise patient stratification [31]. By analyzing their unique molecular and clinical profiles in precision medicine, SHAP helps explain why specific treatments might work better for certain patients [32,33].

## 2- Method details

### 2−1 - Metabolomics multimodal datasets

The datasets in this study originated from breast cancer tissue samples collected under the METAcancer FP7 project (Denkert et al., 2012; Hilvo et al., 2011). The METAcancer initiative sought to determine whether metabolite alterations could aid in the molecular classification of breast cancer and identify novel prognostic and predictive biomarkers. Metabolomic profiling was conducted across three complementary platforms: High-Resolution Magic Angle Spinning Proton Nuclear Magnetic Resonance (HRMAS ¹H NMR), Gas Chromatography–Time-of-Flight Mass Spectrometry (GC-TOFMS), and Liquid Chromatography–Mass Spectrometry (LC-MS). While the original METAcancer datasets provided valuable single-platform insights, such as cancerous vs. non-cancerous tissue and ER status, the present study focused on cross-platform integration to investigate the HER2 subtype specifically. S1 Table, provides a summary of sample counts and metabolite features across the three platforms [34–37]

### 2−2 - Data curation

Three distinct metabolomics platforms—NMR, GC-MS, and LC-MS—were used to profile metabolites, with particular emphasis on breast cancer subgroups including HER2. Earlier large-scale efforts, such as the METAcancer FP7 project, were unable to achieve classification within the HER2 group; this study was designed to address those challenges through rigorous data integration. Datasets were curated to retain only samples with confirmed hormone receptor and HER2 status across all platforms. This refinement resulted in a harmonized cohort of 253 samples, subsequently balanced using oversampling to 423 per platform to ensure equal representation. Feature counts varied across platforms: 180 for NMR, 161 for GC-MS, and 183 for LC-MS positive mode. LC-MS negative mode was excluded due to the limited number of features (34 known metabolites). The resulting dataset preserved the unique characteristics of each platform while enabling robust cross-platform integration for downstream analyses.

NMR preprocessing included peak assignment for the identification of known metabolites using established chemical shift libraries and reference standards. To reduce peak overlap and improve spectral resolution, Statistical Total Correlation Spectroscopy Editing (STOCSY-E) was applied, exploiting correlation patterns across the spectral domain to align peaks arising from the same metabolite. This enhanced annotation and alignment ensure consistency across samples.

Metabolite intensities from all platforms were normalized using conventional approaches. No explicit batch-effect correction was applied in our analysis, and it remains unclear whether any was performed during initial preprocessing. Nevertheless, the consistently high performance and stability across platforms suggest that any potential batch-related effects were likely minimal.

### 2-3 Data preprocessing: Missing labels, normalization, and scaling

A small proportion of missing metabolite intensity values was identified in the dataset. Although excluding the affected samples would have been a justifiable approach, we preferred to retain the entire cohort and applied Multiple Imputation by Chained Equations (MICE) [38] to estimate the missing values, thereby preserving statistical power and avoiding potential bias. Data preprocessing was then tailored to each modeling framework. For Multiple Kernel Learning (MKL), features from each platform were rescaled to the range [0,1], followed by row-wise normalization to satisfy ‖Xᵢ‖²₂ = 1. Multi-view kernels were subsequently generated for the LC-MS, GC-MS, and NMR datasets, with kernel normalization applied before integration. In the Deep Transfer Learning framework, each platform was independently standardized to zero mean and unit variance before feature extraction via a custom autoencoder incorporating L1/L2 regularization, dropout, and batch normalization. The encoded representations were then rescaled and used as inputs to the ANN classifier. For concatenation-based, ensemble, and Deep Forest approaches, features were standardized using z-score scaling, and concatenated datasets were additionally subjected to row normalization to mitigate scale discrepancies across platforms. In single-platform analyses, features were both standardized and row-normalized to ensure comparability of metabolite intensities and facilitate consistent downstream interpretation.

### 2-4 Machine learning framework and implementation

All machine learning analyses were performed using Python 3.6. The following methodologies are described in detail, with relevant references provided to clarify their application and support reproducibility.

### 2-5 Oversampling

Considering the significant disparity in the distribution of HER2 positive (0.1462) and HER2 negative (0.8538) instances across all three platforms, oversampling was implemented using the SMOTE (Synthetic Minority Over-sampling Technique) library in Python to rectify this discrepancy. This strategy indirectly augmented the sample sizes, yielding 423 instances for each platform, thus promoting a more balanced dataset [39].

### 2-6 Feature selection

The differential abundance of HER2 positive versus HER2 negative instances across all three platforms did not present significant disparities compared to Cancerous versus Non-cancerous, making differential expression feature selection methods such as fold change and effect size unsuitable for this study (see supplement, S1 Fig). Consequently, four algorithmic feature selection techniques—ReliefF, SVM-RFE, RF-RFE, and Mutual Information—were evaluated and compared to determine the optimal method for feature elimination. A brief overview of each method is provided below:

**ReliefF (Relief Feature Selection):** ReliefF is a filter-based feature selection method that gauges feature relevance by assessing their ability to distinguish instances of the same and different classes. It assigns weights to features based on their impact on classification accuracy [40].

**SVM-RFE (Support Vector Machine Recursive Feature Elimination):** SVM-RFE is a wrapper-based feature selection technique employing Support Vector Machines (SVMs) in a recursive elimination process. It iteratively removes the least important features, ranking them based on their contribution to SVM performance [41].

**RF-RFE (Random Forest Recursive Feature Elimination):** RF-RFE is a wrapper-based feature selection method utilizing Random Forests. It recursively eliminates less important features by assessing their impact on the accuracy of a Random Forest classifier. Features are ranked based on their contribution to model performance [42].

**Mutual Information:** Mutual Information is a filter-based feature selection measure quantifying the dependency between two variables. It assesses the information shared between each feature and the target variable, ranking features based on their information content and relevance [43].

The effectiveness of feature selection methods was evaluated using the SVM-Linear model with C = 1. The optimal number of features for each method on individual platforms was determined. In this study, stability measures, such as the Jaccard score, were employed to assess the consistency of feature selection methods across various data subsets. The Jaccard score quantifies the similarity among selected features across different subsets. A higher Jaccard score indicates greater stability, signifying that the feature selection method is more reliable.

### 2-7 Individual platform classification

The feature selection method employed consistently across all platforms was SVM-RFE. For binary classification within the HER2 group, three SVM models—SVM-linear, SVM-RBF, and SVM-poly—were utilized. Each dataset underwent partitioning into training (80%) and testing (20%) sets, treated as independent data subsets. Repeated stratified cross-validation was employed to enhance model robustness, accompanied by a grid search for hyperparameter optimization. To mitigate overfitting, the range of C values was restricted between 0.01 and 20. Evaluation metrics for individual and integrated multimodal models included accuracy, F1 score, balanced accuracy, sensitivity, specificity, and Matthew’s correlation coefficient (MCC), all reported with 95% confidence intervals (CI 95%). MCC was particularly informative in the context of imbalanced datasets, providing a comprehensive assessment by accounting for both false positives and false negatives.

### 2-8 Integrated multimodal techniques

Multimodal data integration was achieved using several methodologies, including concatenation ensemble, multiple kernel learning (MKL), and deep transfer learning.

#### 2-8-1 Straightforward concatenation and concatenation ensemble methods.

Three distinct metabolomics platforms were horizontally concatenated, aligning samples in rows and featuring distinct datasets as columns. Individual platform features underwent selection using SVM-RFE. Each dataset was scaled, normalized, and oversampled before concatenation. For the concatenation-ensemble method, base model training involved a grid search for hyperparameter tuning of the SVM polynomial kernel after splitting the data. Optimal hyperparameters were identified, including the regularization parameter (C) and polynomial degree. Subsequently, a Stacking Classifier was created, combining SVM polynomial kernel, Random Forest (RF), and XGBoost (XGB) models. In the Bagging Classifier, an RF bagging classifier was the base estimator. Logistic Regression was employed as the final estimator/meta-learning in the stacking model, introducing L2 regularization (penalty = ’l2,’ C = 1.0) and implementing 5-fold cross-validation during the stacking process.

Model performance for both concatenated and concatenation-ensemble approaches was rigorously evaluated using nested repeated stratified cross-validation, providing robust hyperparameter tuning and controlling model complexity. Performance metrics—including accuracy, F1 score, balanced accuracy, sensitivity, specificity, and MCC—were reported along with CI 95%, ensuring a statistically reliable assessment of model generalization.

#### 2-8-2 Cascade deep forest.

In this study, the Deep Forest model was employed to classify and evaluate data from three distinct metabolomics platforms, each of which had undergone feature selection. These platforms were horizontally concatenated, aligning samples in rows and representing different datasets as columns. The Deep Forest model utilized a Cascade Forest framework, composed of multiple layers of forest estimators, including both Random Forest and Extra Trees classifiers. This hierarchical structure enabled the progressive refinement of feature representations across layers. Hyperparameter tuning focused on key parameters such as the number of layers, forests per layer, and estimators per forest.

Initially, we experimented with a multi-grained scanning approach using a sliding window. However, this led to higher risks of overfitting and lower evaluation metrics. Consequently, the multi-grained scanning was excluded from the final workflow.

Model evaluation and hyperparameter optimization were conducted using repeated stratified cross-validation. This approach ensured a robust and reliable framework for the classification and analysis of complex metabolomics datasets.

#### 2-8-3 Multiple kernel learning.

EasyMKL, a scalable Multiple Kernel Learning (MKL) algorithm, was adeptly employed for integrating information from multiple platforms. The algorithm’s scalability and computational efficiency enable effective management of many kernels [44,45]. To optimize the MKL framework, the ‘cvxopt’ solver, renowned as a quadratic programming solver, was utilized to identify the optimal combination of kernels. The combined kernel matrix was split into training and testing sets, employing 5-fold repeated stratified cross-validation. Hyperparameter tuning for λ (the regularization parameter controlling the trade-off between model complexity and accuracy) and C (the cost parameter penalizing misclassification) was conducted via grid search within the MKL framework. 95% confidence intervals (CI 95%) were estimated using bootstrap resampling to provide a robust measure of model performance uncertainty.

#### 2-8-4 Deep transfer learning / Multimodal deep learning.

The autoencoders employ a carefully designed architecture featuring customized encoding dimensions, ‘Tanh’ activation functions, and optimization using the Adam optimizer with mean squared error, which has proven pivotal [36].

This approach sets the stage for feature extraction from three distinct platforms. Features from these platforms are concatenated and then partitioned into a 70% training and 30% testing split. Similarly, the ANN adopts a comparable architecture, utilizing the ‘tanh’ activation function and the Adam optimizer. Autoencoders and ANN undergo fine hyperparameter tuning to mitigate overfitting and bolster performance. This tuning is facilitated by a nested repeated stratified cross-validation K-Fold approach with five splits, ensuring thorough model evaluation and decreasing the risks of overfitting.

Hyperparameters are optimized using GridSearchCV from scikit-learn. This method samples hyperparameters from predefined distributions, and model performance is evaluated through cross-validation. Additionally, Gaussian noise is added to the training data to reduce overfitting, and models are reinitialized during cross-validation to ensure robustness.

Within the autoencoder framework, hyperparameter optimization encompasses various facets:

- Encoding dimension: Governing the size of the bottleneck layer and compression levels.
- Batch size: Influencing the speed of convergence and memory utilization.
- Dropout rate: A regularization technique aimed at mitigating overfitting.
- L1 and L2 penalties: Regularization mechanisms designed to discourage large parameter values.
- Learning rate: Pivotal for adjusting weights during optimizer training.

In the ANN domain, optimization extends beyond basic parameters like batch size, dropout rate, and learning rate. It includes ‘alpha,’ which determines the strength of L2 regularization, thereby shaping the model’s capability to curb overfitting by penalizing large weights. the number of artificial neurons directly influencing the model’s aptitude to grasp intricate patterns. Model performance was evaluated with CI 95% estimated via bootstrap resampling, providing a robust assessment of prediction reliability.

#### 2-8-5 Feature weights, feature mapping, and selecting important features.

The process begins by aligning feature names to match the input dimensions of the encoder’s bottleneck layer weights, ensuring consistency in naming. Next, feature mapping is performed by extracting the weights of the bottleneck layer and identifying the top contributing input features for each encoded feature based on the absolute values of their weights. This creates a mapping of encoded features to their most influential input features and weights. Using this mapping, the importance of the global feature is calculated by aggregating the absolute weights of each input feature across all encoded features. The aggregated values are then ranked to identify the most essential features globally, providing insights into feature significance for individual platforms [46,47].

#### 2-8-6 SHapley Additive exPlanations (SHAP) analysis.

The process begins with splitting the dataset. Features are then scaled to ensure uniformity in input data. Next, a **Random Forest** model is optimized through hyperparameter tuning using GridSearchCV, and the best model is selected. Feature selection is performed using Recursive Feature Elimination (RFE) to identify the most relevant features for the model. The refined model is retrained on these selected features. Subsequently, **SHAP** values are computed to quantify feature contributions for predictions, separated by classes (e.g., HER2+ and HER2-). Mean absolute SHAP values are calculated for each feature per class, enabling comparison of feature contributions. A data frame is created to rank features by importance and visualize the top contributors.

### 2-9 Model validation and overfitting prevention

Several strategies were employed to minimize the risk of overfitting during model development. Model performance was evaluated using cross-validation and train–test partitioning to ensure consistency across folds. In datasets with class imbalance, oversampling was applied to equalize class representation. For most models, repeated nested stratified cross-validation was implemented to provide stable and unbiased estimates of predictive performance, separating hyperparameter optimization from performance evaluation. For more computationally intensive methods, such as Multiple Kernel Learning (MKL), repeated stratified cross-validation was employed to balance efficiency with robust performance estimation. To quantify the uncertainty of model predictions, 95% confidence intervals (CI 95%) were estimated via bootstrap resampling, providing a probabilistic range for performance metrics such as accuracy, F1 score, and AUC. Additionally, permutation testing of the ROC curve was conducted to assess the statistical significance of classification performance, confirming that observed AUCs were unlikely due to chance. To control model complexity and reduce variance, regularization techniques (L1 and L2 penalties) were applied, and in deep learning models, dropout layers and batch normalization were incorporated to stabilize training and further mitigate overfitting. Performance metrics were systematically compared across folds to detect potential instability.

To further minimize bias introduced by platform sensitivity differences, each dataset (LC-MS, GC-MS, NMR) was independently scaled and normalized before integration, ensuring that no single platform disproportionately influenced the analysis. Feature extraction through autoencoders reduced modality-specific variation, while SHAP-based interpretability identified metabolites with consistent contributions across platforms.

Beyond statistical validation, the biological interpretability of selected features was evaluated. Metabolites were analyzed in the context of known metabolic pathways and cancer biology, ensuring that the models were not only statistically robust but also biologically meaningful.

## 3- Results

### 3−1- Feature selection

Four different methodologies were employed and evaluated to determine the most efficient feature selection method for the HER2 subtype group across various platforms: ReliefF, SVM-RFE, RF-RFE, and Mutual Information. For the assessment, SVM-Linear was used without any hyperparameter tuning (S2 Table). The supporting information (S1 Fig) provides the number of optimized features for each method on individual platforms.

### 3−2 Individual modal classification

The evaluation metrics for three models—SVM-Linear, SVM-RBF, and SVM-Polynomial—across individual platforms are presented in S3 Table. In the NMR dataset, SVM-Linear demonstrates superior performance. Conversely, SVM-RBF outperforms Accuracy, F1 Score, and Specificity in the GC-MS dataset. However, SVM-Linear exhibits higher metrics in Balanced Accuracy, AUC, and MCC, highlighting its overall effectiveness compared to other methods. In the LC-MS dataset, SVM-Polynomial demonstrates superior performance, with higher metrics and lower standard deviations, indicating greater robustness.

### 3−3 Multimodal data integration

The study examined various multimodal techniques, including Straightforward Concatenation, Concatenation-Assembled, Deep Forest, Multiple Kernel Learning, and Deep Transfer Learning. The evaluation employed datasets, namely NMR, GC-MS, and LC-MS, which featured uniform sample quantities (matching rows) but differed in the number of features (distinct columns).

#### 3-3-1 Straightforward concatenation.

**Fig 1:**
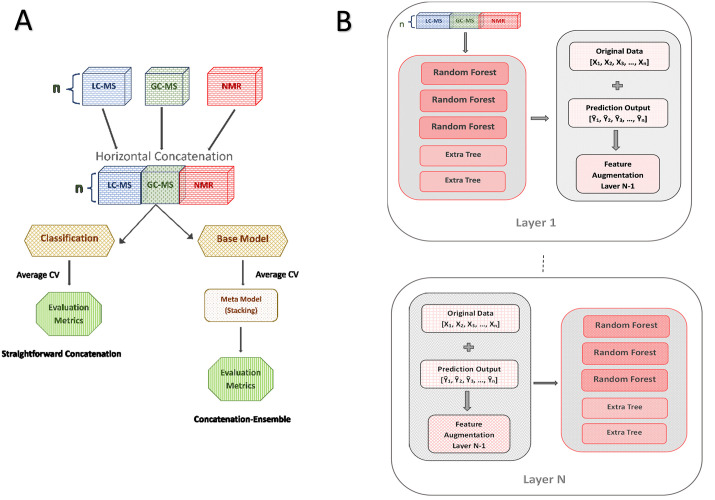
Workflows for concatenation, ensemble, and cascade deep forest. **A)** Straightforward Concatenation and Concatenation-Ensemble: In the Straightforward Concatenation approach, datasets are directly merged, followed by nested-stratified cross-validation and evaluation using SVM models. In the Concatenation-Ensemble approach, a base model combining SVM-poly, Random Forest (RF), and XGBoost (XGB) is trained on the validation set to generate predictions, which are then used to train a meta-model for final evaluation. **B)** Cascade Deep Forest: Datasets are input into cascade levels where Random Forests and Extra Trees independently generate probabilistic predictions. The final output integrates probabilities across all layers using an average-probabilities strategy.

Datasets were horizontally concatenated while preserving identical label groups, such as HER2-positive and HER2-negative, as illustrated in the workflow shown in **Fig 1A** (right panel). S3 **and** S4 Tables provide a detailed summary of the evaluation metrics for the concatenation method utilizing Repeated Nested Stratified cross-validation. The SVM-RBF model consistently achieved higher overall metrics compared to SVM-Linear and SVM-Poly. While SVM-Poly exhibited greater sensitivity, its specificity was slightly lower than that of SVM-RBF and SVM-Linear.

#### 3-3-2 Concatenation – Ensembled.

The horizontally concatenated datasets were used to construct an ensemble model incorporating SVM-Poly, Random Forest (RF), and XGBoost as base learners. Hyperparameter tuning was performed using grid search, while Logistic Regression served as the meta-learner. Both stacking and bagging strategies were applied during model training. To reduce the risk of overfitting, Repeated Nested Stratified Cross-Validation was employed. The overall workflow of the Concatenation–Ensemble approach is illustrated in **Fig 1A**. S4 Table presents the evaluation metrics, showing that the Concatenation–Ensemble approach achieved consistently better performance than the straightforward concatenation method across multiple criteria.

#### 3-3-3 Deep forest.

The workflow of the Deep Forest model is illustrated in **Fig 1B**. The Cascade Deep Forest model has a hierarchical structure with multiple layers, where each layer integrates both Random Forest and Extra Trees classifiers to enhance the model’s performance. Initially, the original dataset—comprising concatenated multimodal metabolomics data with prior feature selection—is processed by several forest models in the first layer, producing class probability predictions.

Each layer of the Deep Forest model generates probabilistic predictions that are combined with the original features and passed iteratively through subsequent layers. Final probabilities from all forests are aggregated to produce the classification outcome, allowing the model to progressively refine data representation and enhance robustness. S4 Table shows that the Deep Forest achieved high and balanced performance (Accuracy and AUC), with slightly higher specificity but lower sensitivity than the Concatenation–Ensemble model. Overall, both models performed similarly, with Deep Forest favoring specificity and the ensemble approach offering a more balanced trade-off.

#### 3-3-4 Multiple kernel learning.

**Fig 2:**
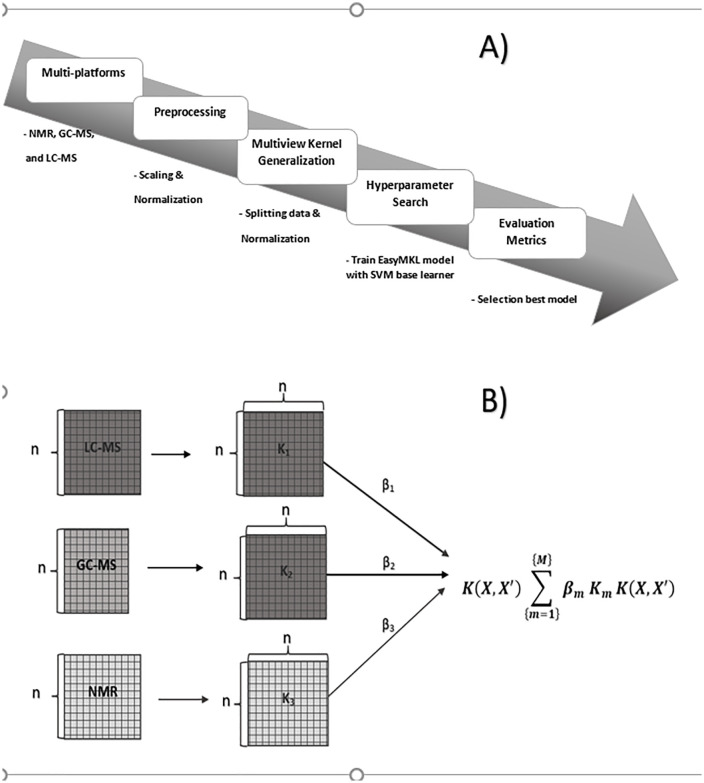
The Multiple Kernel Learning Process. **A)** The figure outlines key stages, including the transition from individual platforms to MKL creation, hyperparameter tuning, and subsequent model evaluation. **B)** The integration of three metabolomics platform datasets into kernel matrices is depicted. These matrices are then combined, incorporating corresponding weights (βm), representing a linear combination of a set of n kernels **(K)**.

MKL was implemented using the EasyMKL algorithm, a scalable approach designed to optimize combination parameters for a predefined set of kernel matrices. **Fig 2A** outlines the primary steps involved in MKL models, and **Fig 2B** provides a schematic illustration of the generation of the Multiview kernel matrix. S4 Table summarizes the evaluation metrics for Multiple Kernel Learning (MKL) applied to integrated LC-MS, GC-MS, and NMR datasets. The SVM-Linear model demonstrated robust performance across accuracy, F1 score, AUC, balanced accuracy, sensitivity, specificity, and MCC. Bootstrap confidence intervals for SVM-Linear further confirm the stability and reliability of these results, highlighting the model’s consistent performance in capturing patterns across heterogeneous metabolomics data

#### 3-3-5 Autoencoder transfer learning, extract feature weights, and feature mapping.

**Fig 3:**
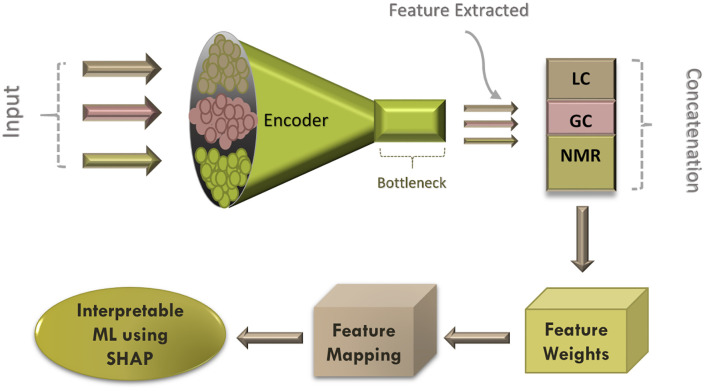
Custom autoencoder transfer learning architecture. The model utilizes three datasets as input, which are processed through an encoder and a hidden layer for dimensionality reduction. Extracted features from individual platforms are either concatenated and used in subsequent classification steps to identify cancer subtypes or assigned weights and subsequently mapped to identify metabolite names and select key features (30 metabolites per platform). Finally, the top 10 most contributory and abundant metabolites are analyzed using the SHAP method.

The architecture of the custom autoencoder is illustrated in **Fig 3**, highlighting the cross-platform feature concatenation process and the extraction of significant features. Input data from the three distinct platforms was compressed into a compact latent space through an efficient encoding process. The input layer progressed sequentially to a hidden layer with a designated encoding dimension, which played a pivotal role in defining the size of the metabolic profile and effectively reducing the dimensionality of input data from the LC-MS, GC-MS, and NMR platforms. Features from all three platforms were horizontally concatenated to form a multiview dataset for subsequent classification.

In parallel, features extracted from the bottleneck of the encoder for each platform were assigned feature weights. These weights facilitated the identification of significant metabolites, with the top 30 metabolites selected from each platform based on their respective weights. To identify specific metabolite names, features were mapped to detect key metabolites according to their weights. The SHapley Additive exPlanations (SHAP) method was subsequently applied to evaluate the contributions and abundance of these selected metabolites, offering insights into their relevance in the classification process.

#### 3-3-6 Interpretable autoencoder feature extraction using SHapley Additive exPlanations (SHAP).

**Fig 4:**
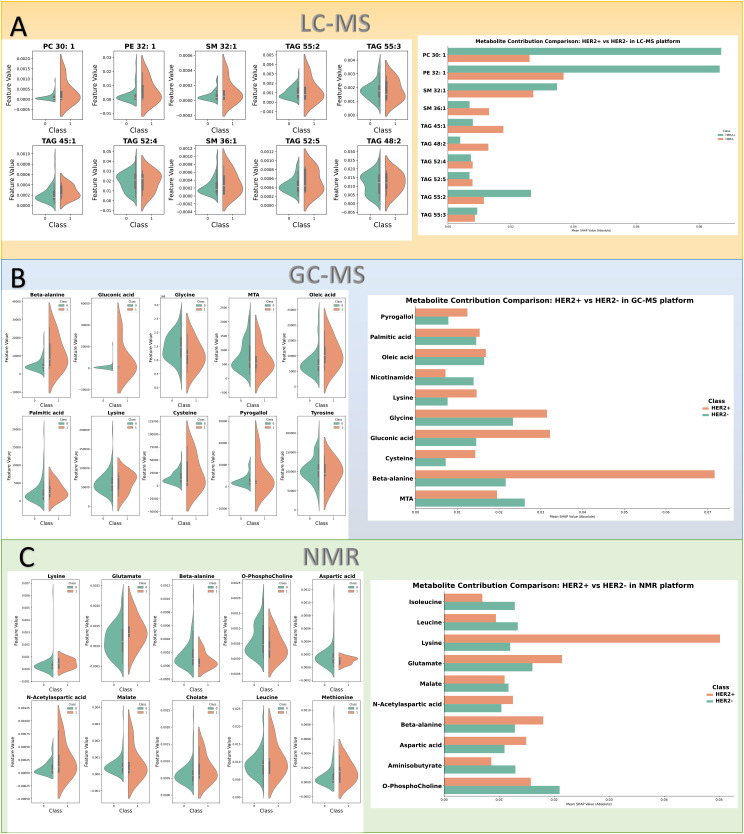
SHAP-based violin plot analysis of metabolite abundance and feature contributions distinguishing HER2 status across three metabolomics platforms. The left panels show violin plots of the top 10 SHAP-ranked metabolites, where the width indicates the density of data points across feature values, enabling visualization of abundance differences between HER2-positive (salmon-orange) and HER2-negative (teal-green) subtypes. The right panels in each platform present the corresponding mean absolute SHAP values, reflecting each metabolite’s relative contribution to distinguishing between the two groups. Results are shown for **A)** LC-MS, **B)** GC-MS, and **C)** NMR platforms.

SHAP analysis was conducted using a random forest model on 30 significant metabolites identified across each platform to assess the absolute contributions and abundances of the top 10 metabolites. To ensure a more focused metabolite analysis for the HER2 subtype, ER- or PR-positive samples were excluded, and only HER2-enriched samples were classified as HER2-positive. S2 Fig depicts the distribution of these top features, and **Fig 4** (right panel) presents a comparative analysis of absolute contributions across the three datasets. Within the LC-MS platform, distinct differences were observed between HER2+ and HER2- samples, with mono-unsaturated phospholipids such as PC 30:1, PE 32:1, and SM 32:1 showing elevated levels in HER2 + samples. Triacylglycerols (TAGs) displayed varying patterns: TAG 45:1, TAG 48:2, and TAG 55:3 were reduced in HER2 + samples, while TAG 52:5, TAG 55:2, TAG 58:10, and TAG 60:2 showed increased levels. In the GC-MS platform, higher contributions were observed in HER2 + samples for beta-alanine, glycine, oleic acid, gluconic acid, cysteine, and lysine, whereas 5’-deoxy-5’-methylthioadenosine (MTA) and nicotinamide had more significant contributions in HER2- samples. The NMR analysis indicated elevated levels of glutamate, methionine, cholate, N-acetyl aspartic acid, aspartic acid, beta-alanine, and lysine in HER2-positive samples. In contrast, isoleucine, leucine, o-phosphocholine, glycerophosphocholine, glutamine, and myoinositol demonstrated increased contributions in HER2-negative samples. **Fig 4** (left panel for each platform) illustrates the comparative abundance of the top 10 metabolites across all platforms. In HER2-positive samples, increased levels were observed for PC 30:1, PE 32:1, SM 32:1, SM 36:1, TAG 55:2, TAG 55:3, TAG 52:4, TAG 52:5, TAG 50:4, gluconic acid, beta-alanine, oleic acid, cysteine, tyrosine, and pyrogallol. Conversely, nicotinamide, arabinose, palmitic acid, MTA, Cer 42:2, cytidine-5-diphosphate, and TAG 48:2 were more abundant in HER2-negative samples.

**Fig 5:**
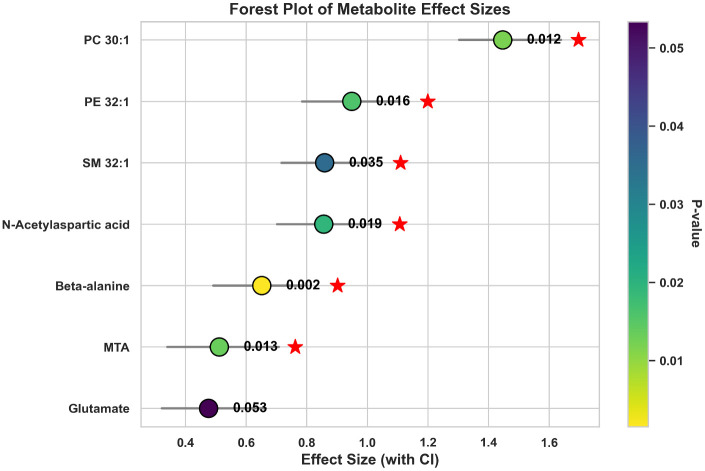
Forest plot of effect sizes for selected metabolites. The horizontal axis represents the effect size with 95% confidence intervals (CI) indicated by horizontal lines. Each metabolite is represented by a colored dot at its effect size, with the corresponding p-value labeled adjacent to the point. A red asterisk () denotes statistical significance (p < 0.05).

This study focuses on advanced machine learning techniques for biomarker discovery and interpretation, with SHAP providing biological insights. To complement these methods and account for the limited number of significant metabolites, statistical validation was applied using thresholds of absolute effect size (Cohen’s d > 0.5) and t-test p-value < 0.05, as shown in **Fig 5**. The final panel included PC 30:1, PE 32:1, SM 32:1, β-alanine, MTA, and N-acetylaspartic acid, with glutamate as a borderline candidate.

#### 3-3-7 Classification using artificial neural network.

**Fig 6:**
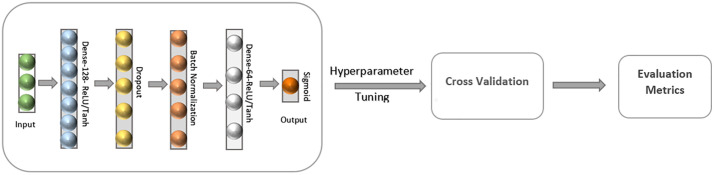
The architecture of the ANN designed for autoencoder concatenated data. The subsequent results underwent hyperparameter tuning and cross-validation using a repeated 5-fold stratified cross-validation approach.

The schematic workflow of the sequential Artificial Neural Network (ANN) model applied to the concatenated dataset is shown in **Fig 6**. The model features an input layer that integrates data from three distinct platforms, followed by a dense layer consisting of 128 **nodes.** Subsequently, a dropout layer is introduced to mitigate overfitting and enhance generalization, followed by batch normalization, a critical technique for stabilizing and accelerating the training process. A dense layer with 64 nodes is added to extract hierarchical features, utilizing Tanh activation functions to capture intricate details within the multimodal data. Finally, the output layer is tailored for binary classification methods, comprising a single node activated by the sigmoid function. All hyperparameters are fine-tuned through repeated nested stratified cross-validation.

The ANN-based deep transfer learning model demonstrated outstanding performance and strong consistency across training, cross-validation, and independent test sets (**Table 1**). On the independent test set, it achieved an accuracy of 0.9925, an F1 score of 0.9925, an AUC of 0.9958, a sensitivity of 1.0000, and a specificity of 0.9850.

**Table 1:** Performance metrics of the ANN-based deep transfer learning model. Metrics are reported for training (fold-level) and cross-validation (fold-level), along with the final model performance on the independent test set. Bootstrap confidence intervals (CI) for the test set are provided to assess model stability. Reported metrics include Accuracy, F1 Score, AUC, Balanced Accuracy, Sensitivity, Specificity, and Matthews Correlation Coefficient (MCC). Note: Test AUC permutation p-value = 0.0000.

Metric	Training (Fold-level)	Cross-validation (Fold-level)	Final Model (Test Set)	Bootstrap CI (Test Set)
Accuracy	0.9996 (0.9986–1.0005)	0.9934 (0.9874–0.9995)	0.9923	0.9925 (0.9769–1.0000)
F1 Score	0.9996 (0.9987–1.0005)	0.9935 (0.9876–0.9995)	0.9924	0.9925 (0.9748–1.0000)
AUC	1.0000 (1.0000–1.0000)	1.0000 (1.0000–1.0000)	0.9957	0.9958 (0.9856–1.0000)
Balanced Acc.	0.9996 (0.9986–1.0005)	0.9934 (0.9874–0.9995)	0.9923	0.9925 (0.9758–1.0000)
Sensitivity	1.0000 (1.0000–1.0000)	1.0000 (1.0000–1.0000)	1.0000	1.0000 (1.0000–1.0000)
Specificity	0.9992 (0.9973–1.0011)	0.9869 (0.9748–0.9990)	0.9846	0.9850 (0.9516–1.0000)
MCC	0.9992 (0.9973–1.0010)	0.9871 (0.9752–0.9990)	0.9847	0.9851 (0.9543–1.0000)

Transfer deep learning was applied for ER-status prediction to evaluate model consistency. The model showed highly consistent performance across training, cross-validation, and test sets. On the test set, it achieved an accuracy of 0.9828 (95% CI: 0.9569–1.0000), F1-score of 0.9825 (95% CI: 0.9533–1.0000), AUC of 0.9970 (95% CI: 0.9908–1.0000), and MCC of 0.9656 (95% CI: 0.9131–1.0000), confirming strong generalization (S5 Table).

#### 3-3-8 A comprehensive comparison of integration multimodal.

**Fig 7:**
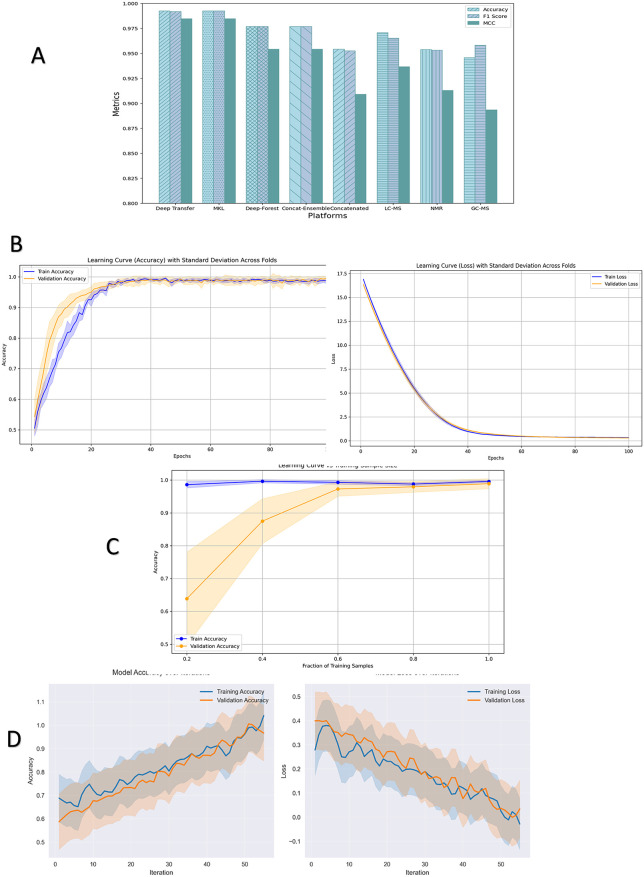
Performance evaluation and learning dynamics of predictive models. **A)** Comparison of accuracy, F1 score, and MCC across integrated multimodal approaches versus individual platforms, highlighting top-performing models such as Deep Transfer Learning and MKL for their consistently superior performance. **B)** Learning curves showing accuracy and loss during training and cross-validation in the deep transfer learning model. **C)** ANN learning curve depicting performance saturation with increasing training fraction. Validation accuracy rises and plateaus, while training accuracy remains high. Converging curves and narrowing standard deviations indicate minimal overfitting and sufficient sample size for robust generalization across the heterogeneous metabolomics dataset. **D)** MKL learning curves illustrating model performance across iterations. Accuracy for training and validation steadily approaches maximum, with loss decreasing toward zero. Shaded regions represent standard deviation across runs, reflecting some variability in performance despite overall positive trends.

A comprehensive comparison of Accuracy, F1 Score, and MCC across integrated multimodal approaches and individual platforms is presented in **Fig 7A and** S6 Table. To facilitate comparison, the top-performing model from each method was selected. The Deep Transfer Learning and MKL approaches demonstrated outstanding performance across all metrics. The Concatenated Ensemble and Deep Forest ranked second, showing strong performance and confirming their competitiveness as integrated multimodal solutions. Among individual platforms, LC-MS displayed particularly high accuracy and F1 Scores, especially when evaluated using 95% bootstrap confidence intervals within nested stratified cross-validation, indicating excellent performance. The Concatenated platform also achieved competitive results, reflecting average performance across LC-MS, GC-MS, and NMR datasets. **Fig 7B** illustrates the learning curves for accuracy and loss during training and cross-validation for the ANN-based deep transfer learning model. **Fig 7C** depicts performance saturation of the ANN as a function of training sample fraction. Consistency between cross-validation and training metrics was excellent when more than 60% of the samples were used. Validation accuracy increased sharply with sample fraction and plateaued with full dataset utilization, indicating sufficient sample size for robust generalization. Near-perfect training accuracy across all fractions, along with converging curves and shrinking standard deviations, confirmed minimal overfitting and effective handling of inter-platform variability. **Fig 7D** illustrates the overall learning dynamics of the MKL model, with the accuracy curve approaching ~1.0 and the loss converging toward zero over 50 iterations. Notably, the relatively wide standard deviation bands throughout training indicate considerable variability in performance across runs, highlighting potential instability in the kernel combination process.

#### 3-3-9 Average and grand effect size of the individual platform.

**Table 2** presents the average and grand effect sizes for each platform. Notably, all platforms demonstrate effect sizes exceeding 0.5, with accuracies surpassing 80%. This finding aligns with prior research suggesting that a small to moderate sample size can be sufficient for analysis when using high-quality datasets. Among the platforms, GC-MS stands out with significantly higher average and grand effect sizes (greater than 2) than the others.

**Table 2:** Average and Grand effect size of individual platforms.

No		Average Effect size	Grand Effect size
1	**LC-MS**	0.565	0.572
2	**GC-MS**	2.075	2.641
3	**NMR**	0.571	0.575

## 4- Discussion

In the present study, the optimal feature selection method was meticulously chosen based on the superior performance demonstrated by the SVM-linear technique across four specified methods (ReliefF, SVM-RFE, RF-RFE, and Mutual Information). Notably, SVM-RFE exhibited exceptional performance on LC-MS and NMR datasets, whereas comparable performance was observed across all methods, except RF-RFE, in GC-MS data. To ensure simplicity and consistency, we standardized the feature selection approach across all platforms, selecting SVM-RFE as the preferred method. Before initiating comparisons, we carefully fine-tuned the optimal number of features for each method on individual platforms.

Transfer deep learning was implemented using a custom autoencoder that took three metabolomics modalities as input, with dimensionality reduction performed in the encoder’s hidden layer. Features extracted from the encoder’s bottleneck were analyzed using feature weights, generating feature maps identifying reduced-dimensional features. The top 30 metabolites from each analytical platform were selected based on this analysis. Subsequent SHAP analysis refined this selection to the top 10 metabolites for each platform (S2 Fig). The SHAP analysis, which compared contributions and abundance values between HER2-positive and HER2-negative samples in the LC-MS platform, revealed elevated levels of mono-unsaturated phospholipids—specifically PC 30:1, PE 32:1, and SM 32:1—in HER2-positive samples. This elevation corresponds to increased Stearoyl-CoA Desaturase-1 (SCD1) expression in HER2-positive breast cancer cells, particularly in cases negative for estrogen receptor (ER) and progesterone receptor (PR) [48–50]. SCD1 plays a crucial role in breast cancer by influencing membrane fluidity and promoting cancer cell proliferation and survival. It facilitates the conversion of saturated fatty acids to monounsaturated fatty acids, essential for maintaining membrane integrity and supporting tumor growth [51–53]. Triacylglycerol (TAG) species showed distinct patterns in their contributions and abundances. Specifically, TAG 55:2, TAG 52:5, and TAG 58:10 were elevated in HER2-positive samples, reflecting increased lipid droplet formation and fat storage in cancer cells. Conversely, TAG 48:2, TAG 50:4, and TAG 55:3 levels were decreased in HER2-positive samples, likely due to enhanced TAG consumption during tumor cell division and proliferation. In GC-MS analysis, palmitic acid (C16:1) and oleic acid (C18:1) showed increased contributions and abundances due to enhanced SCD1 activity. These fatty acids, synthesized via SCD1-mediated pathways, are crucial precursors for monounsaturated phospholipids such as PC 30:1 and PE 32:1. Elevated gluconic acid levels have been identified in HER2-positive breast cancer. As a byproduct of glucose oxidation, gluconic acid plays a pivotal role in cellular metabolism and is frequently dysregulated in cancer cells. Increased levels of gluconic acid are linked to altered glycolysis and heightened oxidative stress in breast cancer models. Furthermore, gluconic acid can serve as an alternative carbon source, providing cancer cells with metabolic flexibility that supports their survival and proliferation. In the context of breast cancer, this elevation in gluconic acid may indicate the metabolic reprogramming that underlies cancer development and progression [54–56]. In addition, the GC-MS dataset reveals a significant increase in the contribution and abundance of β-alanine in HER2-positive breast cancer. Elevated levels of β-alanine may reflect a compensatory endogenous mechanism that counteracts tumor-induced oxidative stress and sustains tumor growth through aberrant metabolic pathways. This finding underscores its role in the metabolic reprogramming of amino acids, supporting the enhanced glucose utilization and energy metabolism required for rapid cancer cell proliferation [57–60]. Interestingly, a plausible hypothesis suggests that β-alanine’s potential as an anticancer agent in breast cancer is multifaceted, depending on whether it is endogenous or exogenous. Elevated endogenous β-alanine may aid tumor survival by facilitating metabolic reprogramming and managing oxidative stress. In contrast, exogenous β-alanine supplementation could shift this balance toward anticancer effects by increasing carnosine levels beyond the tumor’s metabolic control. This increase may interfere with glycolysis, buffer intracellular acidity, and selectively elevate oxidative stress in cancer cells while sparing normal tissues. In addition, carnosine’s capacity to modulate key oncogenic pathways, including PI3K/AKT/mTOR and MAPK signaling, together with its potential role in epigenetic regulation, may further hinder tumor progression and enhance the effectiveness of immune-based therapies [50,61–67]. These combined effects may collectively disrupt the metabolic demands and invasive capacity of HER2-positive tumor cells, positioning exogenous β-alanine as a promising therapeutic adjunct in this aggressive cancer subtype. Nevertheless, further studies are needed to analyze the effects of β-alanine as a supplement or therapeutic agent in this context.

Nicotinamide levels and contributions are significantly decreased in HER2-positive breast cancer. Previous studies suggest that nicotinamide, combined with non-steroidal anti-inflammatory drugs (NSAIDs), can bind to and inhibit the SIRT1 deacetylase. This enzymatic inhibition activates the anti-proliferative p53/p21 pathway, reducing the risk of invasive breast cancer subtypes, including HER2-positive and triple-negative cases [67,68]. The observed low levels of nicotinamide in HER2-positive breast cancer may serve as a potential therapeutic biomarker, guiding strategies to mitigate the aggressiveness of this cancer subtype.

In addition, 5’-methylthioadenosine (MTA) shows reduced contribution and abundance in HER2-positive tumors compared to HER2-negative tumors. MTA has demonstrated potential as an anti-tumor agent by inhibiting tumor cell proliferation and invasion while promoting apoptosis. These effects are linked to MTA’s ability to regulate the inflammatory microenvironment of tumors, potentially creating conditions that are less conducive to cancer progression [69–71]. Furthermore, in the NMR platform, elevated levels of lysine, N-acetylaspartic acid, O-phosphocholine, and cholate were observed in HER2-positive breast cancer. The increase in lysine and glutamate are particularly associated with the reprogramming of amino acid synthesis, and N-acetyl aspartic acid, O-phosphocholine, and cholate are linked to promoting tumor growth, metastasis, cell proliferation, and enhanced cancer survival. Based on a defined effect size and p-value threshold, statistical analysis identified a subset of six metabolites—PC 30:1, PE 32:1, SM 32:1, β-alanine, MTA, and N-acetylaspartic acid—across all platforms. While these metabolites underscore the significance of these biomarkers, further investigation using SHAP analysis is prioritized to identify additional biomarker signatures. S3 Fig provides a schematic overview of HER2-driven signaling and metabolic reprogramming, informed by KEGG pathways and published literature. It illustrates how HER2 activation engages PI3K/AKT/mTOR and MAPK/ERK signaling, leading to SREBP1-mediated induction of SCD1, enhanced mono-unsaturated fatty acid synthesis, and lipid membrane biosynthesis. Moreover, alterations in glutamine and β-alanine metabolism are consistent with the metabolomic signatures identified in this study.

Evaluation of individual binary classifications within the HER2 group applied SVM-RFE feature selection for SVM models, revealing distinct performance characteristics. To improve robustness, hyperparameter tuning was conducted using grid search, repeated stratified cross-validation, and L2 regularization, effectively mitigating overfitting and enhancing generalization. In NMR datasets, the SVM-Linear model exhibited higher performance, achieving notable accuracy, sensitivity, and MCC values. Conversely, in GC-MS data, SVM-RBF excelled in accuracy and F1 score, while SVM-Linear demonstrated superiority in sensitivity and AUC. Across all platforms, LC-MS emerged as the top performer, achieving remarkable accuracy, F1 score, sensitivity, and MCC through an SVM polynomial.

Straightforward concatenation resulted in accuracy, F1 score, AUC, and specificity that were either higher or equivalent to those observed in the GC-MS and NMR datasets. However, compared to LC-MS, the performance was considered average. The Concatenation-Ensemble and Deep-Forest methods consistently outperformed the individual datasets across all metrics. Employing Easy-MKL for SVM models in Multiple Kernel Learning demonstrated outstanding performance, highlighting robust capabilities in classification tasks. Specifically, SVM-RBF and SVM-Poly demonstrated remarkably high accuracy, F1 score, AUC, and MCC. Additionally, SVM-Linear displayed robust overall performance, particularly excelling in sensitivity and specificity. These findings support previous studies, indicating enhanced performance of multimodality compared to individual platforms [22,26]. Complementing these results, the Multimodal Deep Learning approach—using a tailored autoencoder architecture to capture latent representations from each platform and subsequently integrating them within the ANN classifier—further elevated performance. Beyond predictive accuracy, this framework enabled the extraction of informative metabolites across LC-MS, GC-MS, and NMR datasets, providing both improved classification and biologically meaningful insights through a strategy that distinguishes it from conventional multimodal integration methods. The comprehensive comparison of integrated multiview metabolite performance revealed that MKL and Deep Transfer Learning secured the top positions across all performance metrics, providing a well-balanced estimation. The concatenation-ensemble and Deep Forest methods ranked second, exhibiting relatively strong evaluation metrics. The analysis of learning curves for accuracy and loss, along with the evaluation metrics for training, testing, and cross-validation in Deep Transfer Learning, reveals a remarkable level of consistency. On the other hand, MKL exhibits a high average consistency between training and cross-validation learning curves. However, the wide standard deviation observed across folds indicates notable variability in performance between runs, suggesting that while overall trends are stable, individual iterations can fluctuate considerably. Comparisons between the training and testing datasets demonstrate a strong correlation, reinforcing the model’s robustness despite this variability. Bootstrap 95% confidence intervals were calculated for SVM-Linear models in the EasyMKL framework, demonstrating stable performance. CI 95% were excluded for SVM-RBF and SVM-Poly, as EasyMKL in high-dimensional settings with these kernels can produce unstable estimates. In such cases, bootstrap resampling or nested stratified cross-validation may not reliably capture performance variability due to increased complexity and sensitivity to hyperparameters.

The identification of integration methods requiring optimal computational resources poses significant challenges. While the concatenation ensemble typically relies on simpler operations, such as concatenation and basic ensemble techniques, adopting optimization methods for each model increases computational complexity. In Deep Forest, multiple layers of decision trees are trained, resulting in moderate computational complexity. However, Deep Forest tends to have higher computational complexity and longer running times than concatenated-ensemble methods due to the need to train and evaluate multiple layers of decision trees iteratively. Furthermore, in Concatenation-Ensemble, Deep-Forest, and MKL methods, individual platform features are initially selected before integration. In Deep Transfer, features are specified using an autoencoder, followed by an artificial neural network (ANN) classification, indicating a moderately complex computational process. When applied for feature extraction, autoencoders provide a compact data representation that reduces complexity compared to more elaborate methods. However, achieving good generalization and optimizing hyperparameters can require substantial computational resources. The robustness of these approaches significantly enhances the accuracy and reliability of determining HER2 status, thereby improving precision outcomes. Achieving a sensitivity of 1 in MKL and Deep Transfer Learning may suggest a potential risk of overfitting, particularly in the context of oversampling. However, this performance primarily reflects the accurate classification of the HER2-negative majority class, with no false negatives observed, rather than overfitting to minority samples. High consistency across training, testing, and cross-validation phases supports reliable generalization. Additionally, the techniques such as nested repeated stratified cross-validation, L2 regularization, and controlled oversampling mitigate overfitting risks, ensuring robust and stable model evaluation. Due to the relatively small size of each platform dataset (423 samples for individual datasets after oversampling), which is often considered insufficient for Deep Learning methodologies, this study acknowledges a recognized phenomenon observed in prior research where ML accuracies tend to stabilize after a certain number of samples in sufficiently high-quality data. This study evaluated dataset quality by considering average and grand effect sizes of data, focusing specifically on those with effect sizes greater than 0.5 and accuracies exceeding 80% for robust classification [14]. Moreover, although larger sample sizes are generally advantageous in high-dimensional domains such as genomics or imaging, where they help mitigate overfitting, metabolomics may allow for meaningful analysis even with comparatively smaller cohorts. This is due to the intrinsic nature of metabolomic data, which reflects the end products of cellular processes, combined with the benefits of precise feature selection and the inherent biological relevance of metabolites. Integrated multimodal metabolomics using MKL has shown strong performance and potential for cancer detection. However, MKL presents challenges in feature analysis, as composite features from multiple platforms complicate the interpretation of distinct metabolite signatures. To address this challenge, we applied autoencoder-based feature extraction for each platform, followed by SHAP analysis to evaluate feature contributions. This framework highlights the dual role of deep transfer learning as a high-performance classification tool and as a means to identify biologically meaningful biomarkers.

Restricting the analysis to metabolomics reduced data heterogeneity, enabling a more rigorous evaluation of methodological robustness. The framework established here can be extended to multi-omics integration, where transcriptomics, proteomics, or other data layers provide complementary biological insights. Such integration requires either matched samples across platforms or advanced computational strategies—such as multi-head autoencoders or shared latent embedding frameworks—that align heterogeneous datasets. This approach parallels our multi-platform metabolomics workflow and supports future efforts to advance precision medicine by offering a more comprehensive view of tumor biology and therapeutic response. Candidate biomarkers identified through computational analyses can be experimentally validated to bridge precision medicine and translational applications. For example, metabolomics findings may be confirmed using targeted LC-MS to detect elevated levels of β-alanine, glutamate, or monounsaturated fatty acids (MUFAs) such as PC 30:0 in HER2-positive breast cancer tissues. Complementary validation in transcriptomics may be performed via RT-qPCR to confirm up- or down-regulated gene expression patterns. Such validation strengthens diagnostic potential and establishes a foundation for therapeutic development, supporting the translation of computational discoveries into clinically actionable strategies.

This study presents a synergistic framework that uniquely integrates multi-platform metabolomics using a custom autoencoder for feature extraction and dimensionality mapping, combined with SHAP-driven interpretability. By extracting bottleneck features from LC-MS, GC-MS, and NMR platforms, quantifying their contributions, and integrating them into a unified framework, the pipeline enables both accurate classification and biologically meaningful biomarker discovery. Unlike other advanced multimodal strategies, which often rely on opaque latent representations or demand extensive computational resources, this approach provides a novel, interpretable, and directly applicable strategy, allowing feature-level insights that reflect underlying biology. This methodology facilitates robust detection of subtype-specific metabolic signatures and exemplifies a unique, translationally relevant application of deep transfer learning in HER2-positive breast cancer.

Despite these advances, certain limitations remain. The relatively modest sample size per platform (423 samples post-oversampling) may limit immediate generalizability. Class imbalances among subgroups and incomplete metadata could influence interpretability, although model performance remained consistently high across cross-validation and test sets. Importantly, the samples analyzed were primarily breast tumor tissues and adjacent non-cancerous tissues, which may not fully represent the metabolic characteristics of normal tissue, and the invasive nature of tissue collection (surgery or biopsy) limits routine clinical applicability. Translating these findings to non-invasive biofluids, such as blood or plasma, is necessary, as metabolite concentrations in these matrices may differ or fall below detectable levels, potentially affecting diagnostic utility. The absence of external validation cohorts or histopathological confirmation further limits independent verification, emphasizing the need for future studies in contemporary or prospective datasets.

From a translational perspective, this multimodal metabolomics framework holds potential for clinical application in breast cancer diagnostics. By integrating LC-MS, GC-MS, and NMR data through a custom autoencoder with SHAP-driven interpretability, the model facilitates the identification of HER2-positive tumors and their associated metabolic signatures within a diagnostic workflow. Coordinated efforts among multidisciplinary teams—including computational scientists, wet-lab researchers, and clinicians—are essential to validate metabolite signatures, standardize protocols, and ensure reliable clinical interpretation. Moreover, incorporating additional omics layers, such as transcriptomics or proteomics, alongside multiple metabolomics platforms could more comprehensively detect biomarkers, enhancing diagnostic accuracy, sensitivity, and potential therapeutic stratification. Future work focusing on prospective cohorts, biofluid validation, histopathological confirmation, and integrated multi-omics approaches will be critical to translate this methodology from research to clinical practice.

## Supporting information

S1 Table Summary of sample counts and metabolite coverage across LC-MS, GC-MS, and NMR platforms. “Initial Sample Count” indicates raw samples before subtype filtering, and “Subtype-Matched Sample Count” shows samples matched across all platforms. After oversampling, 432 samples were used for multiplatform integration. Total Metabolites for NMR includes Known, Unknown, Fragments, and baseline signals.
(DOCX)

S2 Table The evaluation metrics for four different feature selection methods using SVM-linear. The Jaccard score was used to assess feature stability. This score quantifies the similarity among selected features across data subsets. A higher Jaccard score indicates greater stability, meaning the feature selection is more reliable.
(DOCX)

S3 Table A Comparison of Evaluation Metrics for Individual Metabolomic Platforms. Three SVM models—SVM-Linear, SVM-RBF, and SVM-Polynomial—were applied to each platform after feature elimination using SVM-RFE. The evaluation metrics include Accuracy (Acc), F1 Score (F1), AUC Score (AUC), Sensitivity (Sen), Specificity (Spe), Balanced Accuracy (BA), and Matthews Correlation Coefficient (MCC). Note: Test AUC permutation p-value = 0.0000.
(DOCX)

S4 Table Evaluation Metrics for Test Classification of Integrated Metabolomic Platforms. Methods compared include straightforward concatenation with SVMs, concatenation-ensemble with SVMs, RF, and XGB, deep-forest with RF, multiple kernel learning with SVMs, (scratch and pre-trained). Metrics evaluated include Accuracy (Acc), F1 Score (F1), AUC Score (AUC), Sensitivity (Sen), Specificity (Spe), Balanced Accuracy (BA), and Matthews Correlation Coefficient (MCC).Note: Test AUC permutation p-value = 0.0000.
(DOCX)

S5 Table Evaluation metrics of the deep transfer learning–based ANN model for classification of estrogen receptor (ER) status. Results are reported for training, cross-validation, and independent test sets, with metrics including accuracy, F1-score, AUC, balanced accuracy, sensitivity, specificity, and MCC. The consistently high values across evaluation stages highlight the strong predictive performance of the model in distinguishing ER status. Note: Test AUC permutation p-value = 0.0000.
(DOCX)

S6 Table Comparative overview of five multi-platform metabolomics integration methods. Performance is evaluated in terms of predictive accuracy, biological interpretability, computational complexity, and methodological strengths and weaknesses.
(DOCX)

S1 Fig Optimization of the Number of Features for Four Feature Selection Methods. Four Feature Selection Methods — ReliefF, SVM-RFE, RF-RFE, and Mutual Information (MI) — culminate in the determination of the optimal number of features for comparing feature selection methods across individual platforms.
(DOCX)

S2 Fig Distribution of the top 10 metabolites across three defined platforms. Significant metabolites include PC 30:1, PC 32:1, and PE 32:1 in LC-MS; Beta-alanine, Glycine, and Nicotinamide in GC-MS; and Glutamate, Lysine, Aminobutyrate, and Malate in NMR.
(DOCX)

S3 Fig Overview of HER2-driven signaling and metabolism in breast cancer. **(A)**Schematic representation of metabolic reprogramming in HER2-positive breast cancer. HER2 dimerization with HER2, HER3, or EGFR activates PI3K/AKT/mTOR and RAS/RAF/MEK/ERK1/2 signaling cascades, driving downstream transcriptional programs. One key outcome is activation of SREBP1, which induces SCD1 (stearoyl-CoA desaturase 1) expression and promotes monounsaturated fatty acid (MUFA) synthesis, supporting membrane biosynthesis and tumor growth. Concurrently, HER2 signaling enhances glutamine uptake and metabolism via GLS and transaminases (GOT2, GPT2), generating glutamate (Glu) and α-ketoglutarate (α-KG) to fuel the TCA cycle. This metabolic rewiring elevates aspartate (Asp) and β-alanine, reflecting enhanced nucleotide and amino acid biosynthesis.) **(B)** HER2 overexpression activates both the MAPK/ERK1/2 and PI3K–AKT–mTOR–S6K pathways, driving proliferation, survival, and protein translation. These signaling events are closely linked to metabolic reprogramming, where glutamine metabolism contributes to glutamate and aspartic acid pools, nucleotide turnover connects to β-alanine metabolism, and mTOR–SREBP1 signaling induces SCD1-mediated Mono-unsaturated fatty acid (MUFA) synthesis, collectively supporting the biosynthetic demands of HER2-positive tumor cells. **(C)** The metabolic rewiring associated with HER2 signaling includes increased glycolysis, glutaminolysis, and fatty acid biosynthesis. ERK1/2 and mTOR signaling converge on transcriptional and translational regulators, supporting the synthesis of amino acids such as glutamate and aspartic acid, which fuel nucleotide synthesis and sustain rapid cell division. In parallel, mTOR–S6K activation induces SCD1 (stearoyl-CoA desaturase 1), driving monounsaturated fatty acid (MUFA) production and lipid membrane biosynthesis. Elevated β-alanine metabolism further reflects enhanced nucleotide turnover.
(DOCX)
