# Supplementary material for: Unlocking precision diagnostics: A multimodal framework integrating metabolomics with advanced machine learning techniques
Source: PLoS One. 2026 Jun 15;21(6):e0318473. doi: 10.1371/journal.pone.0318473 (PMC13268153; doi:10.1371/journal.pone.0318473)
Supplement: S1 Table — “Initial Sample Count” indicates raw samples before subtype filtering, and “Subtype-Matched Sample Count” shows samples matched across all platforms. After oversampling, 432 samples were used for multiplatform integration. Total Metabolites for NMR includes Known, Unknown, Fragments, and baseline signals. (DOCX) [file pone.0318473.s009.docx]

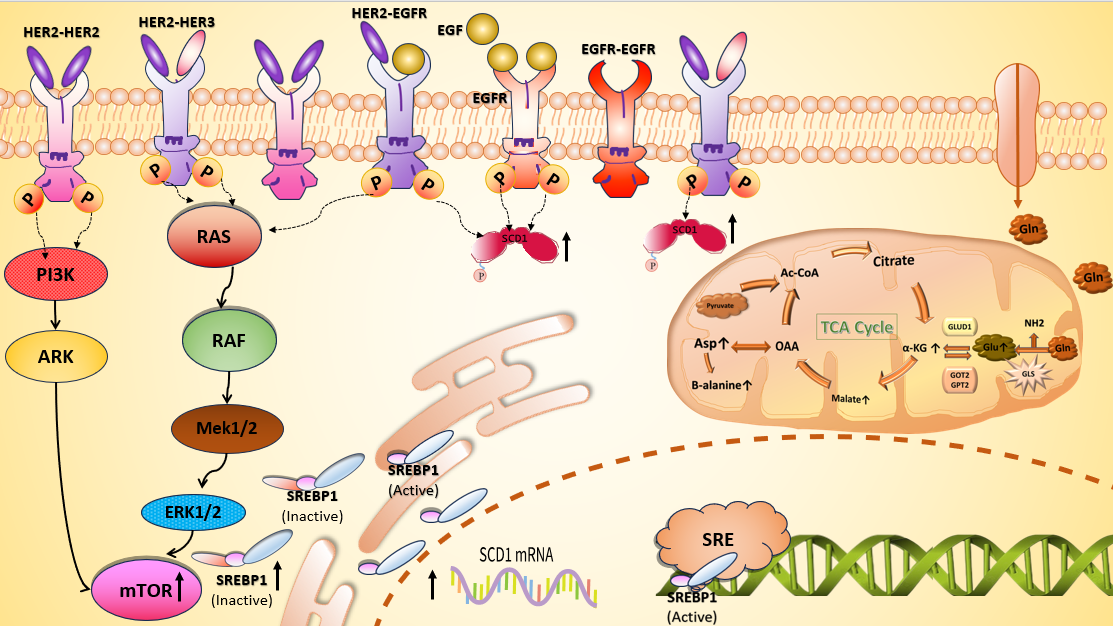

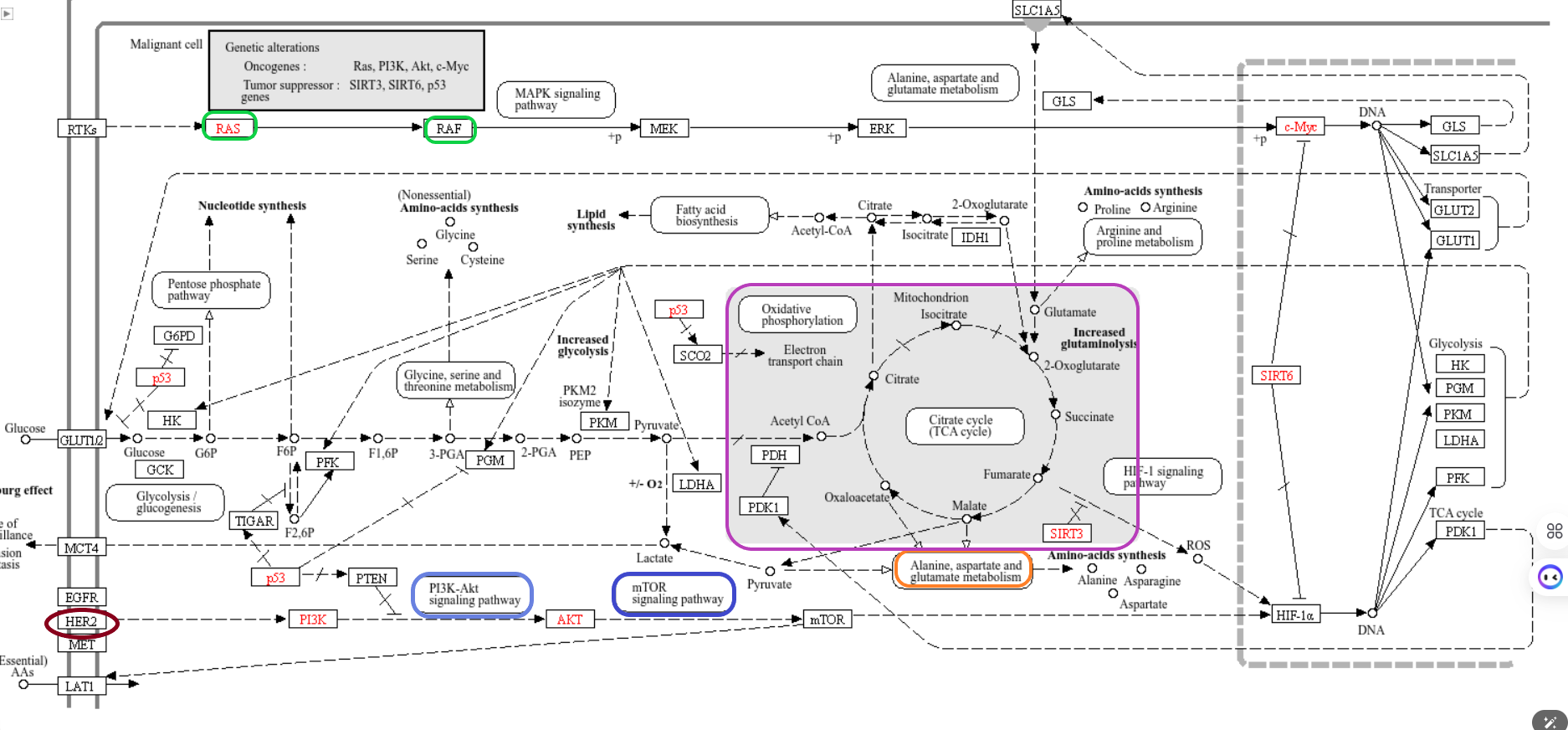


A

B


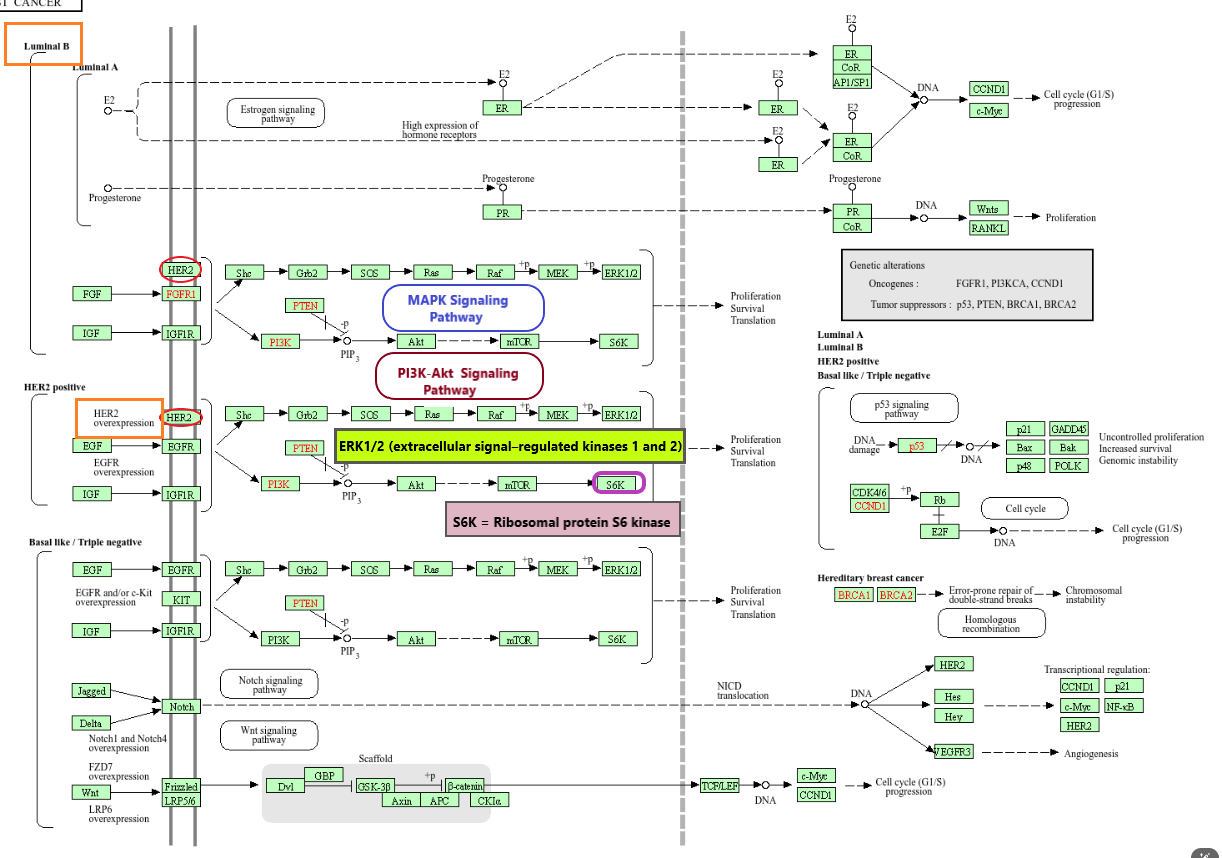


C

**S3 Fig:** **Overview of HER2-driven signaling and metabolism in breast cancer**

**(A)**Schematic representation of metabolic reprogramming in HER2-positive breast cancer. HER2 dimerization with HER2, HER3, or EGFR activates PI3K/AKT/mTOR and RAS/RAF/MEK/ERK1/2 signaling cascades, driving downstream transcriptional programs. One key outcome is activation of SREBP1, which induces SCD1 (stearoyl-CoA desaturase 1) expression and promotes monounsaturated fatty acid (MUFA) synthesis, supporting membrane biosynthesis and tumor growth. Concurrently, HER2 signaling enhances glutamine uptake and metabolism via GLS and transaminases (GOT2, GPT2), generating glutamate (Glu) and α-ketoglutarate (α-KG) to fuel the TCA cycle. This metabolic rewiring elevates aspartate (Asp) and β-alanine, reflecting enhanced nucleotide and amino acid biosynthesis.) **(B)** HER2 overexpression activates both the MAPK/ERK1/2 and PI3K–AKT–mTOR–S6K pathways, driving proliferation, survival, and protein translation. These signaling events are closely linked to metabolic reprogramming, where glutamine metabolism contributes to glutamate and aspartic acid pools, nucleotide turnover connects to β-alanine metabolism, and mTOR–SREBP1 signaling induces SCD1-mediated Mono-unsaturated fatty acid (MUFA) synthesis, collectively supporting the biosynthetic demands of HER2-positive tumor cells. **(C)** The metabolic rewiring associated with HER2 signaling includes increased glycolysis, glutaminolysis, and fatty acid biosynthesis. ERK1/2 and mTOR signaling converge on transcriptional and translational regulators, supporting the synthesis of amino acids such as glutamate and aspartic acid, which fuel nucleotide synthesis and sustain rapid cell division. In parallel, mTOR–S6K activation induces SCD1 (stearoyl-CoA desaturase 1), driving monounsaturated fatty acid (MUFA) production and lipid membrane biosynthesis. Elevated β-alanine metabolism further reflects enhanced nucleotide turnover.
