# Supplementary material for: Unlocking precision diagnostics: A multimodal framework integrating metabolomics with advanced machine learning techniques
Source: PLoS One. 2026 Jun 15;21(6):e0318473. doi: 10.1371/journal.pone.0318473 (PMC13268153; doi:10.1371/journal.pone.0318473)
Supplement: S2 Table — The Jaccard score was used to assess feature stability. This score quantifies the similarity among selected features across data subsets. A higher Jaccard score indicates greater stability, meaning the feature selection is more reliable. (DOCX) [file pone.0318473.s001.docx]

**S1 Table. Summary of sample counts and metabolite coverage across LC-MS, GC-MS, and NMR platforms.** “Initial Sample Count” indicates raw samples before subtype filtering, and “Subtype-Matched Sample Count” shows samples matched across all platforms. After oversampling, 432 samples were used for multiplatform integration. Total Metabolites for NMR includes Known, Unknown, Fragments, and baseline signals.

| Metric | LC-MS | GC-MS | NMR |
| --- | --- | --- | --- |
| Initial Sample Count | 426 | 300 | 355 |
| Subtype-Matched Sample Count | 253 | 253 | 253 |
| Total Metabolites (Known, Unknown) | 426 | 220 | 793 |
| Annotated Metabolites | 210 | 161 | 180 |
| HER2-Negative Samples | 216 | 216 | 216 |
| HER2-Positive Samples | 37 | 37 | 37 |
| ER-Negative Samples | 76 | 76 | 76 |
| ER-Positive Samples | 177 | 177 | 177 |
| Total Samples after oversampling | 432 | 432 | 432 |
