# Supplementary material for: Unlocking precision diagnostics: A multimodal framework integrating metabolomics with advanced machine learning techniques
Source: PLoS One. 2026 Jun 15;21(6):e0318473. doi: 10.1371/journal.pone.0318473 (PMC13268153; doi:10.1371/journal.pone.0318473)
Supplement: S3 Table — Three SVM models—SVM-Linear, SVM-RBF, and SVM-Polynomial—were applied to each platform after feature elimination using SVM-RFE. The evaluation metrics include Accuracy (Acc), F1 Score (F1), AUC Score (AUC), Sensitivity (Sen), Specificity (Spe), Balanced Accuracy (BA), and Matthews Correlation Coefficient (MCC). Note: Test AUC permutation p-value = 0.0000. (DOCX) [file pone.0318473.s002.docx]

**S2 Table: The evaluation metrics for four different feature selection methods using SVM-linear.** The Jaccard score was used to assess feature stability. This score quantifies the similarity among selected features across data subsets. A higher Jaccard score indicates greater stability, meaning the feature selection is more reliable.

|  | Acc | F1 | AUC | Sens | Spe | Balanced Acc | | Jaccard Score |
| --- | --- | --- | --- | --- | --- | --- | --- | --- |
| NMR | | | | | | | | |
| ReliefF | 0.8769 | 0.9036 | 0.8545 | 0.9868 | 0.7222 | | 0.8545 | 0.8242 |
| SVM-RFE | 0.9154 | 0.9325 | 0.8981 | 1 | 0.7963 | | 0.8981 | 0.8736 |
| RF-RFE | 0.8692 | 0.8957 | 0.8506 | 0.9605 | 0.7407 | | 0.8506 | 0.8111 |
| MI | 0.9 | 0.9202 | 0.8823 | 0.9868 | 0.7778 | | 0.8823 | 0.8523 |
| GC-MS | | | | | | | | |
| ReliefF | 0.931 | 0.9474 | 0.9176 | 0.9643 | 0.871 | | 0.9176 | 0.9 |
| SVM-RFE | 0.931 | 0.9474 | 0.9176 | 0.9643 | 0.871 | | 0.9176 | 0.9 |
| RF-RFE | 0.9195 | 0.9391 | 0.9015 | 0.9643 | 0.8387 | | 0.9015 | 0.8852 |
| MI | 0.931 | 0.9474 | 0.9176 | 0.9643 | 0.871 | | 0.9176 | 0.9 |
| LC-MS | | | | | | | | |
| ReliefF | 0.9077 | 0.9268 | 0.8889 | 1 | 0.7778 | | 0.8889 | 0.8636 |
| SVM-RFE | 0.9538 | 0.962 | 0.9444 | 1 | 0.8889 | | 0.9444 | 0.9268 |
| RF-RFE | 0.9077 | 0.9241 | 0.8969 | 0.9605 | 0.8333 | | 0.8969 | 0.8588 |
| MI | 0.8615 | 0.8889 | 0.8441 | 0.9474 | 0.7407 | | 0.8441 | 0.8 |
