# Supplementary material for: Unlocking precision diagnostics: A multimodal framework integrating metabolomics with advanced machine learning techniques
Source: PLoS One. 2026 Jun 15;21(6):e0318473. doi: 10.1371/journal.pone.0318473 (PMC13268153; doi:10.1371/journal.pone.0318473)
Supplement: S4 Table — Methods compared include straightforward concatenation with SVMs, concatenation-ensemble with SVMs, RF, and XGB, deep-forest with RF, multiple kernel learning with SVMs, (scratch and pre-trained). Metrics evaluated include Accuracy (Acc), F1 Score (F1), AUC Score (AUC), Sensitivity (Sen), Specificity (Spe), Balanced Accuracy (BA), and Matthews Correlation Coefficient (MCC).Note: Test AUC permutation p-value = 0.0000. (DOCX) [file pone.0318473.s003.docx]

**S 3 Table:** **A Comparison of Evaluation Metrics for Individual Metabolomic Platforms.** Three SVM models—SVM-Linear, SVM-RBF, and SVM-Polynomial—were applied to each platform after feature elimination using SVM-RFE. The evaluation metrics include Accuracy (Acc), F1 Score (F1), AUC Score (AUC), Sensitivity (Sen), Specificity (Spe), Balanced Accuracy (BA), and Matthews Correlation Coefficient (MCC). Note: Test AUC permutation p-value = 0.0000

|  | Acc | F1 | AUC | Sen | Spe | BA | MCC |
| --- | --- | --- | --- | --- | --- | --- | --- |
| NMR | | | | | | | |
| SVM-Linear | 0.9537 +/- 0.0342 | 0.9533 +/- 0.0334 | 0.9566 +/- 0.0322 | 0.9969 +/- 0.0185 | 0.9164  +/- 0.0622 | 0.9566  +/- 0.0322 | 0.9130 +/- 0.0622 |
| SVM-RBF | 0.9343 +/- 0.0107 | 0.9512 +/- 0.0078 | 0.9100+/- 0.0136 | 0.9943  +/-0.0107 | 0.8258  +/- 0.0274 | 0.100  +/- 0.0136 | 0.8591  +/- 0.0234 |
| SVM-Poly | 0.9167 +/- 0.0218 | 0.9340 +/- 0.0180 | 0.9156 +/- 0.0195 | 0.9193 +/- 0.0304 | 0.9119  +/- 0.0208 | 0.9156  +/- 0.0195 | 0.8225  +/- 0.0447 |
| GC-MS | | | | | | | |
| SVM-Linear | 0.9429 +/- 0.0366 | 0.9431 +/- 0.0348 | 0.9466 +/- 0.0345 | 0.9988  +/- 0.0124 | 0.8944  +/- 0.0659 | 0.9466  +/- 0.0345 | 0.8935  +/- 0.0658 |
| SVM-RBF | 0.9457 +/- 0.0076 | 0.9580 +/- 0.0059 | 0.9394 +/- 0.0093 | 0.9614  +/- 0.0086 | 0.9174  +/- 0.0184 | 0.9394  +/- 0.0093 | 0.8816  +/- 0.0167 |
| SVM-Poly | 0.9444 +/- 0.0183 | 0.9518 +/- 0.0137 | 0.9287 +/- 0.0218 | 0.9832  +/- 0.0159 | 0.8742  +/- 0.0322 | 0.9287  +/- 0.0218 | 0.8790  +/- 0.0404 |
| LC-MS | | | | | | | |
| SVM-Linear | 0.9588 +/- 0.0319 | 0.9585 +/- 0.0307 | 0.9615 +/- 0.0298 | 1.0000  +/- 0.0000 | 0.9231  +/- 0.0569 | 0.9615  +/- 0.0298 | 0.9225  +/- 0.0576 |
| SVM-RBF | 0.9601 +/- 0.0121 | 0.9691 +/- 0.0094 | 0.9557 +/- 0.0141 | 0.9711  +/- 0.0128 | 0.9403  +/- 0.0256 | 0.9557  +/- 0.0147 | 0.9132  +/- 0.0263 |
| SVM-Poly | 0.9708 +/- 0.0166 | 0.9652 +/- 0.0129 | 0.9652 +/- 0.0176 | 0.9848  +/- 0.0181 | 0.9455  +/- 0.0261 | 0.9652  +/- 0.0176 | 0.9366  +/- 0.0363 |
