# Supplementary material for: Unlocking precision diagnostics: A multimodal framework integrating metabolomics with advanced machine learning techniques
Source: PLoS One. 2026 Jun 15;21(6):e0318473. doi: 10.1371/journal.pone.0318473 (PMC13268153; doi:10.1371/journal.pone.0318473)
Supplement: S5 Table — Results are reported for training, cross-validation, and independent test sets, with metrics including accuracy, F1-score, AUC, balanced accuracy, sensitivity, specificity, and MCC. The consistently high values across evaluation stages highlight the strong predictive performance of the model in distinguishing ER status. Note: Test AUC permutation p-value = 0.0000. (DOCX) [file pone.0318473.s004.docx]

**S4 Table: Evaluation Metrics for Test Classification of Integrated Metabolomic Platforms.**

Methods compared include straightforward concatenation with SVMs, concatenation-ensemble with SVMs, RF, and XGB, deep-forest with RF, multiple kernel learning with SVMs, (scratch and pre-trained). Metrics evaluated include Accuracy (Acc), F1 Score (F1), AUC Score (AUC), Sensitivity (Sen), Specificity (Spe), Balanced Accuracy (BA), and Matthews Correlation Coefficient (MCC).Note: Test AUC permutation p-value = 0.0000

| Metrics | Acc | BA | F1 | AUC | Sen | Spe | MCC |
| --- | --- | --- | --- | --- | --- | --- | --- |
| Straightforward Concatenation | | | | | | | |
| SVM-Linear | 0.9540  (0.9540 – 0.9540) | 0.9552  (0.9552–0.9552) | 0.9524  (0.9524– 0.9524) | 0.9552  (0.9552– 0.9552) | 0.9756  (0.9756– 0.9756) | 0.9348  (0.9348-0.9348) | 0.9089  (0.9089-0.9089) |
| SVM-RBF | 0.9425  (0.9425-0.9425) | 0.9403  (0.9403-0.9403) | 0.9367  (0.9367-0.9367 | 0.9403  (0.9403-0.9403) | 0.9024  (0.9024-0.9024) | 0.9783  (0.9783-0.9783) | 0.8864  (0.8864-0.8864) |
| SVM-Poly | 0.9425  (0.9425-0.9425) | 0.9443  (0.9443-0.9443) | 0.9412  (0.9412-0.9412) | 0.9418  (0.9418-09418) | 0.9756  (0.9756-0.9756) | 0.9130  (0.9130-0.9130) | 0.8872  (0.8872-0.8872) |
| Concatenation- Ensemble and Deep Forest | | | | | | | |
| SVM-Poly, RF, and XGB | 0.9777  (0.9425, 1.0000) | 0.9788  (0.9459, 1.0000) | 0.9767  [0.9383, 1.00] | 0.9788  [0.9459, 1.00] | 1.0000  (1.00-1.00) | 0.9565  (0.9565, 0.9565) | 0.9563  [0.8908, 1.00] |
| Multiple Kernel Learning | | | | | | | |
| SVM-Linear | 0.9846 | 0.9846 | 0.9848 | 0.9988 | 1.0000 | 0.9692 | 0.9697 |
| SVM-RBF | 0.9923 | 0.9923 | 0.9924 | 0.9998 | 1.0000 | 0.9846 | 0.9847 |
| SVM-Poly | 0.9923 | 0.9923 | 0.9924 | 1.0000 | 1.0000 | 0.9846 | 0.9847 |
| MKL CI 95% | | | | | | | |
| SVM-linear | 0.9935 [0.9924, 0.9945] | 0.9936  [0.9923, 0.9946] | 0.9936 [0.9923, 0.9946] | 0.9992 [0.9984,0.9999] | 1.0000 [1.00, 1.00] | 0.9871  [0.9848, 0.9894] | 0.9872  [0.9849, 0.9891] |
