# Supplementary material for: Unlocking precision diagnostics: A multimodal framework integrating metabolomics with advanced machine learning techniques
Source: PLoS One. 2026 Jun 15;21(6):e0318473. doi: 10.1371/journal.pone.0318473 (PMC13268153; doi:10.1371/journal.pone.0318473)
Supplement: S6 Table — Performance is evaluated in terms of predictive accuracy, biological interpretability, computational complexity, and methodological strengths and weaknesses. (DOCX) [file pone.0318473.s005.docx]

**S5 Table: Evaluation metrics of the deep transfer learning–based ANN model for classification of estrogen receptor (ER) status.**

Results are reported for training, cross-validation, and independent test sets, with metrics including accuracy, F1-score, AUC, balanced accuracy, sensitivity, specificity, and MCC. The consistently high values across evaluation stages highlight the strong predictive performance of the model in distinguishing ER status. Note: Test AUC permutation p-value = 0.0000.

| Metric/ER | Training (mean ± 95% CI) | Cross-validation (mean ± 95% CI) | Test (Bootstrap CI) |
| --- | --- | --- | --- |
| Accuracy | 0.9981 (0.9958–1.0005) | 0.9833 (0.9736–0.9931) | 0.9828 (0.9569–1.0000) |
| F1-score | 0.9982 (0.9959–1.0005) | 0.9834 (0.9737–0.9930) | 0.9825 (0.9533–1.0000) |
| AUC | 1.0000 (1.0000–1.0000) | 0.9988 (0.9973–1.0003) | 0.9970 (0.9908–1.0000) |
| Balanced Accuracy | 0.9981 (0.9958–1.0005) | 0.9833 (0.9736–0.9931) | 0.9828 (0.9559–1.0000) |
| Sensitivity | 1.0000 (1.0000–1.0000) | 0.9815 (0.9675–0.9954) | 0.9826 (0.9423–1.0000) |
| Specificity | 0.9963 (0.9917–1.0009) | 0.9852 (0.9667–1.0037) | 0.9830 (0.9420–1.0000) |
| MCC | 0.9963 (0.9917–1.0009) | 0.9673 (0.9480–0.9865) | 0.9656 (0.9131–1.0000) |
