# Supplementary material for: Unlocking precision diagnostics: A multimodal framework integrating metabolomics with advanced machine learning techniques
Source: PLoS One. 2026 Jun 15;21(6):e0318473. doi: 10.1371/journal.pone.0318473 (PMC13268153; doi:10.1371/journal.pone.0318473)
Supplement: S1 Fig — Four Feature Selection Methods — ReliefF, SVM-RFE, RF-RFE, and Mutual Information (MI) — culminate in the determination of the optimal number of features for comparing feature selection methods across individual platforms. (DOCX) [file pone.0318473.s006.docx]

**S6 Table: Comparative overview of five multi-platform metabolomics integration methods.** Performance is evaluated in terms of predictive accuracy, biological interpretability, computational complexity, and methodological strengths and weaknesses.

| Attribute / Method | | Concatenation | Concatenation-Ensemble | Deep Forest | MKL | Deep Transfer Learning |
| --- | --- | --- | --- | --- | --- | --- |
| Predictive Performance | Good overall accuracy; lower than advanced methods; | | High; ensemble improves generalization | High; relatively Compatible with heterogeneous multi-platform metabolomic data | Excellent; slightly lower CV/train consistency | Excellent; consistent across train, CV, and test |
| Biological Insight | Weak–Moderate (direct but limited biological insight; lacks integrative insights) | | Low–Moderate; feature-level attribution is indirect and depends on base models in the ensemble. | Low (ensemble nature limits feature-level interpretation) | Moderate (kernel weighting aids interpretability, but kernel matrices limit direct feature mapping) | Moderate-High; latent features capture integrative patterns providing biologically meaningful insights |
| Computational Expenses & Complexity | Low | | Moderate–High (training multiple models and aggregating outputs) | Moderate-high | Highest (kernel matrices + optimization) | High; requires training autoencoder and ANN layers, and latent mapping computations |
| Strengths | Simple implementation; low computational demand | | Robust due to model diversity; better generalization than single models | Effective with tabular/high-dimensional data; | Strong predictive accuracy; integrates multiple data types flexibly | Very good generalization; robust to noise; excellent performance |
| Weaknesses | Fails to capture complex inter-platform relationships; limited biological depth | | Feature rankings unstable; complex workflow; limited direct biological mapping | Limited scalability; restricted biological interpretation | Constructing and optimizing large multi-platform kernel matrices is expensive, nested CV increases computation and variance, which may lead to unstable kernel weight estimation. | Requires careful optimization at each step (autoencoder + ANN + latent mapping) to achieve reliable performance and interpretable outcomes; Outcomes; sensitive to dataset size and for moderate sample size, requires high-quality data for reliable results. |
