# Supplementary material for: Unlocking precision diagnostics: A multimodal framework integrating metabolomics with advanced machine learning techniques
Source: PLoS One. 2026 Jun 15;21(6):e0318473. doi: 10.1371/journal.pone.0318473 (PMC13268153; doi:10.1371/journal.pone.0318473)
Supplement: S2 Fig — Significant metabolites include PC 30:1, PC 32:1, and PE 32:1 in LC-MS; Beta-alanine, Glycine, and Nicotinamide in GC-MS; and Glutamate, Lysine, Aminobutyrate, and Malate in NMR. (DOCX) [file pone.0318473.s007.docx]

**
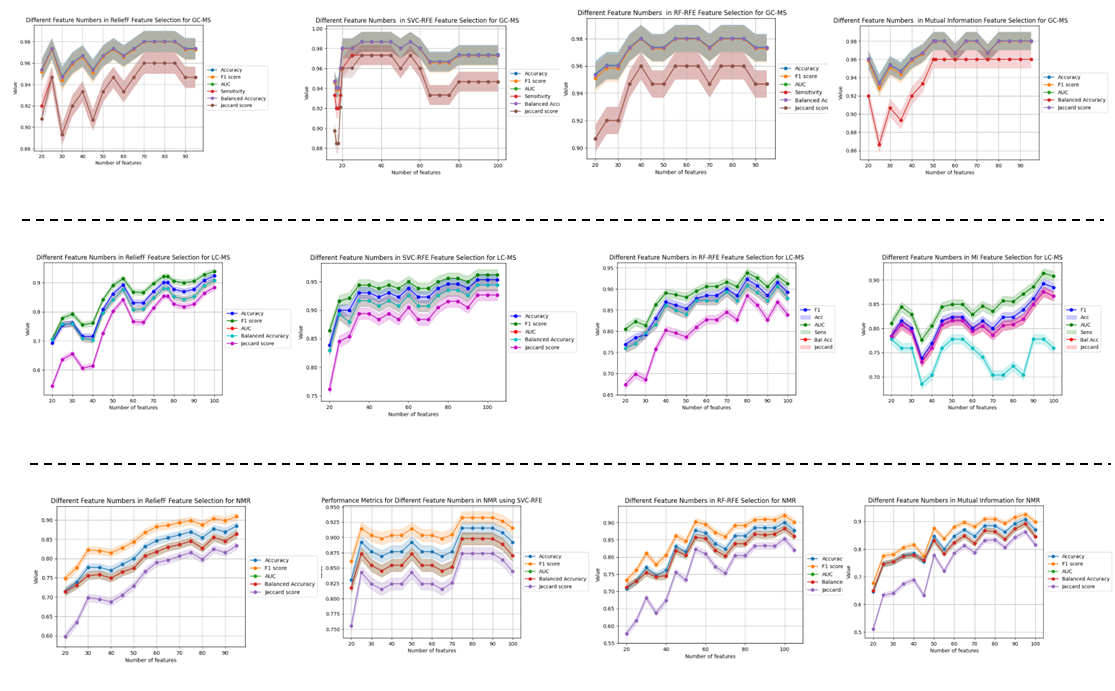
**

**S1 Fig:**  **Optimization of the Number of Features for Four Feature Selection Methods.** Four Feature Selection Methods — ReliefF, SVM-RFE, RF-RFE, and Mutual Information (MI) — culminate in the determination of the optimal number of features for comparing feature selection methods across individual platforms.
