# Supplementary material for: Unlocking precision diagnostics: A multimodal framework integrating metabolomics with advanced machine learning techniques
Source: PLoS One. 2026 Jun 15;21(6):e0318473. doi: 10.1371/journal.pone.0318473 (PMC13268153; doi:10.1371/journal.pone.0318473)
Supplement: Attachment — Submitted filename: plosOne comments.docx [file pone.0318473.s010.docx]

**Unlocking Precision Diagnostics: A Multimodal Framework Integrating Metabolomics with Advanced Machine Learning Techniques**

This manuscript explores integration methodologies of multimodal metabolomics data (LC-MS, GC-MS, NMR) using advanced machine learning (ML) strategies, including Deep Transfer Learning (DTL), Multiple Kernel Learning (MKL), Deep Forest, and ensemble methods. It aims to identify significant biomarkers for HER2-positive breast cancer.

**Major Revisions**

**1. Add external validation cohort**

**Issue**: The study uses only internal cross-validation (nested repeated stratified CV). No independent cohort was used for validation.
**Required Revision**:

- Validate the final models (especially MKL and pre-trained ANN) on an **external metabolomics dataset**, preferably breast cancer samples with HER2 classification.
- If external data is unavailable, apply **leave-one-group-out cross-validation** or **nested LOOCV** as a surrogate.
- Clarify in the methods if any form of **data leakage** was avoided when concatenating features and reusing datasets.

**2. Address overfitting and model complexity**

**Issue**: Exceptionally high metrics (AUC > 0.99, Sensitivity = 1) across all models raise overfitting concerns, especially with a sample size of 253.
**Required Revision**:

- Provide **learning curves with standard deviation bars** across folds.
- Report **confidence intervals** (e.g., 95% CI) for all performance metrics.
- Include **feature distribution plots** to show class separation (e.g., PCA or UMAP of encoded features).

**3. Comparative analysis of biomarkers**

**Issue**: The metabolite panel derived using SHAP is not evaluated as a **biomarker classifier**.
**required revision**:

- Perform **multivariate ROC analysis** using the top 6 statistically significant metabolites (e.g., PC 30:1, PE 32:1).
- Report **AUC, sensitivity, specificity, PPV, and NPV** of this biomarker panel on a **held-out test set**.

**4. Include pathway and network enrichment**

**Issue**: Metabolites are individually discussed but not contextualized in a systems biology framework.
**Required Revision**:

- Use **KEGG Mapper**, **Reactome**, or **MetaboAnalyst** to map significant metabolites to metabolic pathways.
- Include an **enrichment plot** or **network visualization** showing upregulated/downregulated pathways in HER2+.

**5. Clarify sample source and preprocessing**

**Issue**: The sample origin (METAcancer FP7) is not well described.
**Required Revision**:

- Clearly define the **sample types** (e.g., tumor tissue or serum), number per class (HER2+, HER2−), and selection criteria.
- Provide **platform-specific preprocessing steps**, especially normalization, batch correction, and imputation.

**6. Simplify figures and results presentation**

**Issue**: Many figures (especially SHAP and violin plots) are complex and crowded.
**Required Revision**:

- Combine **SHAP bar plots and violin plots** into a single summary figure per platform.
- Move **multi-metric tables** to Supplementary and summarize top results in the main text.

**7. Improve biological interpretation and literature support**

**Issue**: Some claims are speculative or unsupported.
**Required Revision**:

- Avoid speculative claims unless supported by **in vivo or in vitro literature** (e.g., role of β-alanine in HER2+ tumors).
- Cite **relevant mechanistic studies** to support observed metabolite changes (e.g., role of SCD1, gluconic acid in HER2 biology).

**Suggested experimental validation (For Future or Revision Inclusion)**

If the authors have access to lab facilities or collaborators, the following **experimental validations** can be proposed:

**1. Targeted metabolite quantification**

- Use **LC-MS/MS MRM** to quantitatively validate levels of 3–6 key metabolites (e.g., PC 30:1, β-alanine, MTA) in an **independent breast cancer cohort**.

**2. Immunohistochemistry / western blot**

- Evaluate **SCD1 expression** or related enzymes (e.g., FASN, ACC1) in HER2+ vs HER2− breast tumors using IHC or western blot to support lipidomic findings.

**3. Cell line validation**

- Measure metabolite changes in **HER2+ vs HER2− breast cancer cell lines** (e.g., SKBR3 vs MCF7) using targeted metabolomics.

**4. Functional assays**

- Test the effect of **β-alanine or MTA supplementation** on HER2+ cell viability, oxidative stress, or apoptosis to validate their proposed roles.

**8. Integrate a multi-omics comparison or benchmarking**

**Issue**: The study presents multimodal integration but does not benchmark it against other omics layers (e.g., transcriptomics or proteomics).
**Revision**:

- Discuss how the **metabolomics-based integration compares** to existing multi-omics models used in HER2 breast cancer classification.
- Include benchmarking with publicly available **multi-omics data** (like from TCGA-BRCA) if possible, or discuss limitations.

**9. Enhance model interpretability with biological networks**

**Issue**: SHAP values provide feature importance but not biological context.
**Revision**:

- Create a **network diagram** linking top metabolites to **pathways**, enzymes, and relevant genes using tools like Cytoscape or MetScape.
- Highlight connections between **lipid metabolism, amino acid metabolism, and HER2 signaling**.

**10. Incorporate confidence intervals and statistical testing**

**Issue**: Performance metrics lack statistical rigor.
**Revision**:

- Include **95% confidence intervals (CI)** for all reported metrics (Accuracy, F1, AUC, etc.).
- Apply **DeLong’s test** for comparing ROC curves between models.
- Perform **statistical comparison of models** (e.g., t-test or ANOVA) to prove DTL and MKL significantly outperform baseline models.

**11. Justify sample size and power**

**Issue**: Only 253 samples used; sample size justification is weak.
**Revision**:

- Perform **a priori power analysis** or cite **effect size calculations** to justify that the sample size is sufficient.
- Use **learning curves** to show performance saturation i.e., adding more samples doesn’t significantly improve results.

**12. Analyze model robustness to noise**

**Issue**: No robustness analysis is presented.
**Revision**:

- Perform a **noise injection experiment** by randomly permuting a subset of features or labels.
- Report how **robust** each model is to such perturbations. This shows real-world generalizability.

**13. Address batch effects and instrument drift**

**Issue**: Integrating three metabolomics platforms assumes no batch effects.
**Revision**:

- Describe steps taken for **batch effect correction**, alignment, and normalization across LC-MS, GC-MS, and NMR data.
- If possible, run **PCA before and after correction** to show improved integration.

**14. Improve supplementary material quality**

**Issue**: Supplementary figures and tables are disorganized or minimal.
**Revision**:

- Provide complete **data matrices** (anonymized), **model parameters**, **confusion matrices**, and **full SHAP value lists** in supplementary files.
- Include **a GitHub or CodeOcean repository** with full code, model weights, and a Jupyter Notebook for reproducibility.

**15. Add clinical relevance and utility section**

**Issue**: The manuscript lacks practical discussion on translational application.
**Revision**:

- Add a section that discusses **how the model could be translated** into a diagnostic workflow.
- Include limitations such as **cost, scalability, and time** required to run a multimodal ML pipeline in a clinical lab.

**16. Conduct subgroup or stratified analysis**

**Issue**: The HER2 group may contain heterogeneous clinical subtypes.
**Revision**:

- Perform **stratified model performance evaluation** based on menopausal status, tumor grade, or ER/PR status (if available).
- This will improve **personalization** and support precision diagnostics claims.

**17. Explain the role of each ML algorithm choice**

**Issue**: Many ML models are applied, but not all are justified.
**Revision**:

- Provide **a table comparing models**, explaining why each was chosen, its strengths, and what it adds.
- Remove any redundant methods if they don’t add unique value (e.g., if Deep Forest and Ensemble give nearly identical outputs).

**18. Improve the discussion of limitations**

**Issue**: Current limitations are only briefly acknowledged.
**Revision**:

- Explicitly discuss limitations like **class imbalance**, **lack of histopathological validation**, **retrospective data**, and **multi-center variability**.
- A transparent discussion builds credibility.
