# Supplementary material for: Unlocking precision diagnostics: A multimodal framework integrating metabolomics with advanced machine learning techniques
Source: PLoS One. 2026 Jun 15;21(6):e0318473. doi: 10.1371/journal.pone.0318473 (PMC13268153; doi:10.1371/journal.pone.0318473)
Supplement: Attachment — Submitted filename: Reply to reviewersNov25.docx [file pone.0318473.s013.docx]

**Dear Editor-in-Chief and the reviewers,**

We sincerely thank the Editor-in-Chief and the reviewers for their insightful and constructive feedback. We carefully revised the manuscript and implemented changes according to the reviewers' requests wherever possible, while providing reasoned responses in cases where certain suggestions could not be applied directly. To facilitate transparency, we highlighted the revisions within the manuscript:

- **Green highlights** indicate changes made in response to Reviewer #1.
- **Yellow highlights** indicate changes made in response to Reviewer #2.

Below, we provide point-by-point responses to each comment.

**Reviewer #1**

1- **The study only uses internal cross-validation (nested repeated stratified CV). Could the authors validate the final models (especially MKL and pre-trained ANN) on an independent external metabolomics cohort, ideally including breast cancer samples with HER2 classification?** *If external data are unavailable, could alternative strategies, such as leave-one-group-out CV or nested LOOCV be applied? Additionally, please clarify how data leakage was prevented when combining features from multiple platforms and reusing datasets.*

**Response:**

We thank the reviewer for this constructive suggestion, which underscores the importance of robust validation in multimodal machine learning studies. We fully agree that external validation on an independent HER2-annotated cohort with matched LC-MS, GC-MS, and NMR platforms would be ideal; however, despite an extensive search of public repositories (e.g., Metabolomics Workbench, GNPS, TCGA, etc.), no suitable dataset was available. This limitation is common in specialized metabolomics, where the availability of such multi-platform cohort datasets remains scarce.

To address this, we applied repeated stratified cross-validation and nested stratified cross-validation (depending on the model), which provide statistically stable estimates of generalization while balancing class distributions. Compared with leave-one-out CV (LOOCV), these approaches are both more computationally feasible in multimodal integration (avoiding repeated kernel re-computation or full retraining per sample) and less prone to high-variance estimates in moderate datasets (n ≈ 423 post-oversampling). In preliminary tests, LOOCV yielded unstable and inconsistent metrics, whereas stratified CV produced robust and reproducible performance (e.g., test AUC > 0.99 for HER2 classification).

Finally, to prevent data leakage, all preprocessing steps (e.g., normalization, feature selection, autoencoder dimensionality reduction) were performed strictly within training folds of the outer CV loop, with test data held out until final evaluation. Platform features were integrated only after per-fold processing, ensuring complete separation between training and test sets.

**2-The exceptionally high metrics reported (AUC > 0.99, Sensitivity = 1) across all models raise concerns about potential overfitting, particularly given the original sample size of 253. Could you provide:**

- Learning curves with standard deviation bars across folds?
- Confidence intervals (e.g., 95% CI) for all performance metrics?
- Feature distribution plots (e.g., PCA or UMAP of encoded features) to illustrate class separation?

**Response:**

We appreciate the reviewer's valuable feedback. Below, we detail the changes implemented to address the reviewer's requests and improve the clarity of our methods, including the additional analysis requested.

**High AUC values:** For models showing near-perfect AUC (~1), we performed permutation ROC p-value testing, and in all cases, the p-values were effectively 0. This indicates that the observed performance is statistically significant and not attributable to chance.

**Sensitivity:** The apparent sensitivity = 1 observed for HER2 status reflects the dataset composition, where the majority of samples are HER2-negative, resulting in no false negatives.

**Confidence intervals and learning curves:** We have updated all model metrics to include 95% confidence intervals, calculated via bootstrapping or across folds, to provide a robust measure of uncertainty and generalization. In addition, learning curves with standard deviation bars across folds are now included for MKL and Deep Transfer Learning models, demonstrating stable convergence without signs of overfitting.

**Dataset details and oversampling:** After applying oversampling to balance minority classes, the total sample size increased to 423, which improves model training stability and subgroup representation (e.g., HER2-positive, ER-negative). We have updated the Methods section with more detailed information on dataset composition and created a summary table to better illustrate class distributions.

**Feature distribution plots:** We note that PCA and UMAP are unsupervised dimensionality reduction methods and, for complex multi-platform metabolomics data, are best suited for exploratory visualization rather than for validating class separation. Accordingly, we did not rely on these plots to support model performance. Instead, we ensured robust generalization through multiple methodological safeguards, including:

- Data normalization and batch normalization to mitigate platform-specific variation and stabilize training.
- Dropout layers to prevent co-adaptation of neurons and reduce overfitting.
- Hyperparameter tuning via repeated stratified cross-validation, including optimization of network units and dropout rates.
- L1 and L2 regularization to penalize large weights and improve generalization.
- Early stopping to halt training once validation performance plateaued, avoiding overfitting.
- Permutation ROC tests confirming that high AUC values represent the true predictive signal. Together, these measures confirm that the observed high performance—such as sensitivity of 1 for HER2 status —arises from the underlying data structure and rigorous model design, not overfitting or methodological artifacts.

**3-Comparative analysis of biomarkers**
Issue: The metabolite panel derived using SHAP is not evaluated as a biomarker classifier.

- Perform multivariate ROC analysis using the top 6 statistically significant metabolites (e.g., PC 30:1, PE 32:1).
- Report AUC, sensitivity, specificity, PPV, and NPV of this biomarker panel on a held-out test set.

**Response:**

We appreciate the reviewer’s suggestion to evaluate the top SHAP-ranked metabolites as a standalone biomarker panel. In our study, the workflow focused on extracting informative latent features using an autoencoder bottleneck, then applying SHAP analysis to identify the top 30 contributing metabolites across the three platforms, instead of restricting the analysis to a predefined small set of metabolites.

Additionally, in our previously published work in Oct 2024, using the LC-MS lipidomics dataset, we performed ROC and MCC analyses at the individual metabolite level, together with effect size and fold-change assessments under defined p-value thresholds, published here (doi.org/10.1038/s41598-024-71439-7). In the present study, beyond SHAP ranking, we further refined the feature set using statistical filtering (effect size and p-value < 0.05), highlighting six key metabolites. These results are now added, and summarized in a forest plot (Fig. 5, Page 23), which provides a clear visualization of both effect size and statistical significance for these metabolites (previously presented in tabular form).

- **4-Include pathway and network enrichment**
  Issue: Metabolites are individually discussed but not contextualized in a systems biology framework.
  - Use KEGG Mapper, Reactome, or MetaboAnalyst to map significant metabolites to metabolic pathways.
  - Include an enrichment plot or network visualization showing upregulated/downregulated pathways in HER2+.

**Response:**

We agree with the reviewer that contextualizing metabolite-level findings within a systems biology framework is essential, and we’ve added a new figure to reflect this. Our study primarily focused on employing advanced machine learning methods to integrate multi-platform metabolomics data and pinpoint metabolites of translational significance. The reliability of the selected metabolites was supported through comparison with previous studies, rather than relying solely on enrichment and visualization. Nonetheless, in response to the reviewer’s suggestion, we have now included pathway-level information in the Supporting Information. Specifically, we illustrate the HER2 signalling pathway to show how activation of key proteins and enzymes may relate to the observed metabolic alterations. We also schematically present the involvement of monounsaturated phospholipids (e.g., PC 30:1) in HER2-driven SCD1 upregulation, alongside changes in glutamate and β-alanine pathways in HER2-positive cases. These representations complement our machine learning framework and offer mechanistic insights, without being the main basis for metabolite selection (S3 Fig Page 9).

**5-Clarify sample source and preprocessing**

Issue: The sample origin (METAcancer FP7) is not well described.

- Clearly define the sample types (e.g., tumor tissue or serum), number per class (HER2+, HER2−), and selection criteria.
- Provide platform-specific preprocessing steps, especially normalization, batch correction, and imputation.

**Response:**

We thank the reviewer for their attention to this point. We have now expanded the Methods section to provide a clearer description of the sample origin (METAcancer FP7), including the sample types (e.g., tumor tissue or serum), the number of samples per class (HER2-positive, HER2-negative), and other selection criteria as requested (page 7). Additionally, we have summarized the dataset composition, including raw and oversampled samples, in a new table for clarity (S1 Table Page 1). Platform-specific preprocessing steps are now also described in greater detail, covering normalization, batch correction, and imputation procedures, to ensure transparency of the analysis (page 8).

**6- Simplify figures and results presentation**

Issue: Many figures (especially SHAP and violin plots) are complex and crowded.

- Combine SHAP bar plots and violin plots into a single summary figure per platform.
- Move multi-metric tables to Supplementary and summarize top results in the main text.

**Response:**

We thank the reviewer for this suggestion to consolidate the figures. We have now streamlined the figures and results presentation as follows:

- For each platform, we now provide a single summary figure that highlights the top 10 SHAP-ranked metabolites (reduced from the original 15) along with their corresponding violin plots, illustrating both contribution and class-wise distribution (Fig. 4, p. 22).
- Comprehensive multi-metric evaluation tables have been moved to the Supplementary Material, while only the key results are summarized in the main text. Metrics for the ANN-based deep learning model are retained in the main manuscript (Table 1, p. 25).
- The workflows for Concatenation, Ensemble, and Deep Forest approaches have been consolidated into a single schematic (Fig. 1, p. 16).

**7- Improve biological interpretation and literature support**
Issue: Some claims are speculative or unsupported.

- Avoid speculative claims unless supported by in vivo or in vitro literature (e.g., role of β-alanine in HER2+ tumors).Cite relevant mechanistic studies to support observed metabolite changes (e.g., role of SCD1, gluconic acid in HER2 biology).
  Suggested experimental validation (For Future or Revision Inclusion)

**Response:**

We thank the reviewer for stressing the importance of grounding results in biological interpretations from cited literature. In response to the comment, we have made the following change and added additional resources, see new changes/citations  highlighted in yellow in the main text:

- **Expanded literature support:** We have revised the Discussion to include additional mechanistic and clinical studies supporting the roles of the selected key metabolites (e.g., PC 30:1, β-alanine, MTA) in HER2 biology, reinforcing their biological and translational relevance.
- **Targeted metabolite focuses and future plan:** We focus on the key metabolites identified by our workflow and propose targeted metabolite analysis as a future direction for precision medicine and translational research. This includes potential validation in independent cohorts or clinical settings to confirm their utility as biomarkers and to guide therapeutic strategies.

**8- Integrate a multi-omics comparison or benchmarking**
Issue: The study presents multimodal integration but does not benchmark it against other omics layers (e.g., transcriptomics or proteomics).
• Discuss how the metabolomics-based integration compares to existing multi-omics models used in HER2 breast cancer classification.
• Include benchmarking with publicly available multi-omics data (like from TCGA-BRCA) if possible, or discuss limitations.

**Response:**

Although this is a great suggestion, carrying out such tasks is beyond our scope and the available data. While integration with other omics layers (e.g., transcriptomics or proteomics) could further enhance biological insight and benchmarking potential, such integration requires either matched samples across platforms or advanced strategies, such as multi-head autoencoders with shared latent embeddings. These approaches allow weighting and mapping across heterogeneous datasets, analogous to our current multi-platform metabolomics workflow (Page 24).

In the present study, we focused on a single omics type with relatively lower heterogeneity compared to multi-omics datasets. Harmonizing data across multiple omics, particularly when drawn from different sources, remains a key challenge for generalization. We have now highlighted the implementation of multi-omics integration as a future direction in the Discussion section, emphasizing its potential for expanding our framework in precision medicine and translational analysis.

**9**. **Enhance model interpretability with biological networks**
Issue: SHAP values provide feature importance but not biological context.
- Create a network diagram linking top metabolites to pathways, enzymes, and relevant genes using tools like Cytoscape or MetScape.

**Response:**

As described earlier, we have now added a new schematic illustration of the pathways related to the selected metabolites, highlighting connections to key enzymes and relevant biological processes (S3 Fig, Page 9).

**10**. **Incorporate confidence intervals and statistical testing**
Issue: Performance metrics lack statistical rigor.
• Include 95% confidence intervals (CI) for all reported metrics (Accuracy, F1, AUC, etc.).
• Apply DeLong’s test for comparing ROC curves between models.
• Perform statistical comparison of models (e.g., t-test or ANOVA) to prove DTL and MKL significantly outperform baseline models.

**Response:**

We share the same passion and believe in rigor for informatics, analytical, and statistical-based results, and we take further suggestions on board. In our revised analysis, we have included 95% confidence intervals (CI) for all reported performance metrics (Accuracy, F1, AUC, etc.), calculated using bootstrap resampling across folds, which provides a robust estimate of metric variability and model generalization.

For ROC evaluation, we applied permutation-based p-value testing rather than DeLong’s test. Permutation testing is particularly suitable in our setting because it does not rely on distributional assumptions and directly assesses the significance of observed AUC values under the null hypothesis of no predictive signal. In all cases, the permutation p-values were effectively 0, confirming that the high AUCs reflect true predictive performance.

Regarding statistical comparisons between models, conventional tests such as t-tests or ANOVA are not appropriate here, because repeated cross-validation metrics are not independent and may violate test assumptions. Therefore, we focused on reporting confidence intervals and permutation-based significance as more reliable and interpretable measures of model performance.

**11. Justify sample size and power**
Issue: Only 253 samples used; sample size justification is weak.
• Perform a priori power analysis or cite effect size calculations to justify that the sample size is sufficient.
• Use learning curves to show performance saturation i.e., adding more samples doesn’t significantly improve results.

**Response:**

We appreciate the reviewer highlighting this important point, and the points raised are the reasons behind the steps taken to address potential limitations throughout the manuscript. Hence, given the original sample size, we applied SMOTE-based oversampling to address the imbalance in the dataset. SMOTE generates synthetic samples preserving the underlying label-specific patterns. After oversampling, the effective sample size increased to 423 per platform, helping mitigate class imbalance and improve model training stability (S1 Table, Page 1).

In addition, we leveraged transfer learning, which is particularly suitable for generalizing from smaller datasets, allowing the model to learn robust features despite the limited original sample size. We also assessed the quality of each dataset within each platform using Grand and Average effect sizes and accuracy metrics, observing effect sizes greater than 0.5 and accuracy consistently above 80%, indicating sufficient signal for reliable model training.

To further demonstrate sample sufficiency and model stability, we generated learning curves as a function of training sample size. These curves show that model performance saturates as the number of training samples increases, indicating that adding additional samples beyond the current dataset does not substantially improve predictive accuracy. This provides a quantitative justification for the effective sample size used in our study.

**12.** **Analyze model robustness to noise**

Issue: No robustness analysis is presented.

• Perform a noise injection experiment by randomly permuting a subset of features or labels.

• Report how robust each model is to such perturbations. This shows real-world generalizability.

**Response:**

Regarding your comments, in our transfer learning framework, Gaussian noise was incorporated during training to simulate realistic variability in the input features. This was complemented by additional regularization strategies, including L2 weight decay, dropout, and early stopping, as well as rigorous cross-validation. Collectively, these measures enhanced model robustness by reducing overfitting and ensuring that the model’s performance remained stable and generalizable across different datasets.

**13. Address batch effects and instrument drift**
Issue: Integrating three metabolomics platforms assumes no batch effects.
• Describe steps taken for batch effect correction, alignment, and normalization across LC-MS, GC-MS, and NMR data.
• If possible, run PCA before and after correction to show improved integration

**Response:**

All metabolomics datasets (LC-MS, GC-MS, and NMR) were not produced by us but were acquired and preprocessed by some of the top metabolomics labs at the time, normalized and scaled using standard platform-specific procedures to reduce systematic variability (p. 8). Since no batch or run order was available to us, we onboarded a set of approaches to cater for such possibilities. After concatenation, additional normalisations were applied before model prediction to ensure cross-platform comparability. While QC samples were not available to explicitly assess or correct for potential batch effects, the consistently high and reproducible performance across both individual and integrated datasets suggests that any such effects or instrument drift had minimal influence on the outcomes and were not a source of significant variation.

**14. Improve supplementary material quality**
Issue: Supplementary figures and tables are disorganized or minimal.
• Provide complete data matrices (anonymized), model parameters, confusion matrices, and full SHAP value lists in supplementary files.

• Include a GitHub or CodeOcean repository with full code, model weights, and a Jupyter Notebook for reproducibility.

**Response:**

We took the reviewer's suggestion and reorganized the Supplementary Materials to enhance clarity and accessibility. Furthermore, the complete data matrix, confusion matrices, and SHAP value lists are now added to our initially submitted Code Ocean and Figshare repositories, along with the model code, ensuring full transparency and reproducibility.

**15.** **Add clinical relevance and utility section**
Issue: The manuscript lacks practical discussion on translational application.
• Add a section that discusses how the model could be translated into a diagnostic workflow.
• Include limitations such as cost, scalability, and time required to run a multimodal ML pipeline in a clinical lab.

**Response:**

To accommodate review suggestions, we have expanded the discussion to address clinical relevance and utility. The new section outlines how the model could be integrated into a diagnostic workflow following wet-lab validation, considers practical aspects such as cost, scalability, and time, and emphasizes the importance of clinician–researcher collaboration for translation into defined clinical projects (page 34).

**16**. **Conduct subgroup or stratified analysis**

Issue: The HER2 group may contain heterogeneous clinical subtypes.

• Perform stratified model performance evaluation based on menopausal status, tumor grade, or ER/PR status (if available).

**Response:**

To address reviewers’ comments on potential heterogeneity within the HER2-positive group, we have included stratified analysis in the supplementary data. Specifically, the ER status was evaluated using our deep transfer learning model to assess model performance within this clinically relevant subgroup. Stratified analysis allows us to examine whether the model maintains consistent predictive accuracy across different subpopulations, ensuring that a particular subtype does not dominate the reported performance. This approach provides greater insight into the reliability and potential clinical applicability of the model for distinct patient subgroups (S5 Table, page 5).

**17. Explain the role of each ML algorithm choice**

Issue: Many ML models are applied, but not all are justified.

• Provide a table comparing models, explaining why each was chosen, its strengths, and what it adds.

• Remove any redundant methods if they don’t add unique value (e.g., if Deep Forest and Ensemble give nearly identical outputs).

**Response:**

To address the reviewers' suggestions, we have prepared and included a detailed table comparing all applied machine learning models in the supplementary materials. The table outlines the rationale for selecting each model, highlights its strengths, and specifies the unique contributions it provides based on our experimental results and literature (S6 Table, page 6). Because the performance of the Deep Forest and Ensemble models is relatively close, only one representative row is included in the table, as recommended (S4 Table, Page 4).

**18. Improve the discussion of limitations**

Issue: Current limitations are only briefly acknowledged.

• Explicitly discuss limitations like class imbalance, lack of histopathological validation, retrospective data, and multi-center variability.

• A transparent discussion builds credibility.

We are cognizant of limitations in any scenario and in the approach we take. Despite the advances presented, the relatively modest sample size per platform (423 samples post-oversampling) may limit the immediate generalizability of our findings. Potential class imbalances among subgroups and incomplete metadata could also affect interpretability, although model performance remained consistently high across both cross-validation and test sets. Additionally, the absence of external cohorts and histopathological validation restricts independent confirmation, highlighting the need for future studies to validate and extend this framework in larger, multi-centre datasets, hence we add additional text to communicate such limitation (page 35) better.

**Reviewer #2**

1. **Novelty claims:**
   - **Comment:** Although the study uses Deep Transfer Learning (DTL) and Multiple Kernel Learning (MKL) to integrate LC-MS, GC-MS, and NMR metabolomics datasets, the novelty of the work is questioned.
   - **Question/Issue:** How is this work significantly different from existing multimodal and cancer biomarker studies using DTL and MKL?

**Response:**

We appreciate the reviewer’s comment regarding the novelty of our study. While prior research comparing different multimodal integration strategies, including metabolomics, transcriptomics, and proteomics, has focused mainly on review-level discussions or conceptual frameworks, to our knowledge, few studies have implemented practical workflows capable of simultaneously achieving robust cancer subtype classification and identifying biologically significant features. The only closely related work is our previous manuscript, by the same authors, titled *“Uncovering subtype-specific metabolic signatures in breast cancer through multimodal integration, attention-based deep learning, and self-organizing maps”*, which primarily focused on attention-based feature selection and SOM analysis. In contrast, the present study integrates three complementary metabolomics platforms (LC-MS, GC-MS, and NMR) using a custom Deep Transfer Learning (DTL) architecture, extracting latent features through an autoencoder and prioritizing informative metabolites via SHAP-driven interpretability. This methodology enables both accurate HER2-positive breast cancer classification and transparent biomarker discovery, filling a critical gap in existing research. By systematically comparing this approach with alternative multimodal strategies, our study highlights the practical applicability, interpretability, and reproducibility of DTL for high-dimensional metabolomics data, demonstrating its uniqueness as a workflow that moves beyond conceptual or review-level studies (page 35).

2. **Existing literature on multi-omics and metabolomics:**
   - **Comment:** Combining multiple omics modalities in cancer biomarker research is already well established. DTL and MKL have also been used in cancer detection and other biomedical analyses.
   - **Question/Issue:** How does this study improve or go beyond prior work in metabolomics or cancer detection?

**Response:**

We acknowledge that integrating multiple omics modalities for cancer biomarker discovery has been previously explored, and that Deep Transfer Learning (DTL) and Multiple Kernel Learning (MKL) have been applied in certain biomedical contexts. However, most prior work has focused either on conceptual frameworks, review-style comparisons, or limited implementations that prioritize either classification or feature selection, but rarely both simultaneously. Our study advances these approaches in several ways:

1. **Practical multimodal metabolomics implementation**: We integrate three complementary metabolomics platforms (LC-MS, GC-MS, NMR) into a single predictive workflow, rather than performing separate analyses or conceptual comparisons.
2. **Custom Deep Transfer Learning architecture:** Unlike previous studies, our DTL framework uses an autoencoder to extract latent features from high-dimensional metabolomics data, followed by SHAP-driven interpretability to prioritize biologically meaningful metabolites. This enables simultaneous high-performance classification and transparent biomarker discovery, which is rarely achieved in prior studies.
3. **Rigorous comparison framework:** We benchmark DTL against alternative integration strategies, including concatenation, ensemble, and MKL approaches, demonstrating both robustness and mechanistic insight into subtype-specific metabolic signatures.
4. **Clinical and translational relevance:** By identifying HER2-positive breast cancer-specific metabolites across multiple platforms, our study provides actionable insights that extend beyond purely computational performance, supporting future diagnostic and therapeutic applications.

While multimodal integration and DTL/MKL applications do exist, our work uniquely combines practical implementation in metabolomics, with high interpretability, and cross-platform validation to deliver a workflow that simultaneously advances biomarker discovery and accurate subtype classification in metabolomics-based cancer research.

3. **Prior metabolomics findings:**
   - **Comment:** Previous research has identified phospholipids (phosphatidylcholine, phosphatidylethanolamine, sphingomyelin) and amino acids (e.g., β-alanine, N-acetylaspartic acid) as markers in HER2-positive individuals.
   - **Question/Issue:** How do the biomarker results in this study compare to or differ from earlier findings?

**Response:**

We thank the reviewer for raising this point. As discussed in the Results and Discussion sections, our study confirms several previously reported HER2-positive biomarkers, including mono-unsaturated phospholipids (PC, PE, SM) and amino acids such as β-alanine and N-acetylaspartic acid. Where our work extends prior research is in the integrative, multi-platform approach and the SHAP-driven prioritization of features, which allows us to identify these metabolites in the context of cross-platform metabolic signatures rather than single-platform observations. Thus, while the individual biomarkers overlap with previous findings (confirming our data integration works), our study provides robust, reproducible evidence of their subtype-specific relevance across LC-MS, GC-MS, and NMR datasets, highlighting their combined contribution to HER2-positive classification.

4. **Platform differences not discussed:**
   - **Comment:** The manuscript does not critically discuss differences in sensitivity and metabolite coverage across LC-MS/MS, GC-MS, and NMR. LC-MS/MS is more sensitive and detects more metabolites at lower abundance.
   - **Question/Issue:** Are the differences between platforms properly weighted in the integration? Could LC-MS/MS dominate the signal, biasing the results?

**Response:**

We thank the reviewer for this critical point. While LC-MS generally provides higher sensitivity and broader metabolite coverage compared to GC-MS and NMR, our integration framework accounts for platform-specific differences through preprocessing, normalization, and feature scaling. In Multiple Kernel Learning (MKL), each platform generates a separate kernel, which is normalized before integration, ensuring that no single platform disproportionately dominates the combined signal. In Deep Transfer Learning, the custom autoencoder extracts latent representations independently for each platform, followed by SHAP-driven interpretability to weight metabolite contributions relative to classification performance. This strategy allows us to balance the influence of each platform, highlighting biologically relevant metabolites from all datasets rather than reflecting only the most sensitive platform. Therefore, while intrinsic platform differences exist, our methodology mitigates bias and enables robust, multi-platform identification of HER2-positive metabolic signatures.

5. **Methodological justification:**
   - **Comment:** The study applies existing algorithms (DTL, MKL) but does not fully justify or define methodological developments.
   - **Question/Issue:** What methodological innovations are introduced, beyond using existing algorithms?

**Response:**

We appreciate the reviewer’s comment. While DTL and MKL are established algorithms, our study introduces practical methodological innovations. Initially, an optimized feature selection step was implemented to identify subsets of metabolites that maximize classification performance while minimizing redundancy, guiding the input for downstream modeling. A custom autoencoder architecture then extracts latent, platform-specific features from LC-MS, GC-MS, and NMR datasets, which are mapped and interpreted using SHAP-driven analysis to identify biologically meaningful metabolites. Additionally, careful platform-specific normalization and scaling ensure balanced contributions across modalities. The combination of these strategies within a research-ready integration framework allows both robust HER2-positive breast cancer classification and transparent biomarker discovery, distinguishing this work from prior studies that largely remain conceptual or review-focused.

6. **Bias and context:**
   - **Comment:** The sources of bias due to platform sensitivity variation are not directly addressed. Biomarker observations are not contextualized relative to prior results.
   - **Question/Issue:** How are potential biases handled, and how do the findings provide new insights for precision diagnostics?

**Response:**

We thank the reviewer for highlighting this important point. To mitigate potential bias from platform sensitivity differences, each dataset (LC-MS, GC-MS, NMR) was independently scaled and normalized, ensuring that no single platform disproportionately influenced the integrated analysis. Furthermore, feature extraction through a custom autoencoder followed by SHAP-based interpretability allows identification of metabolites whose contributions are robust across platforms. By contextualizing the observed biomarkers—such as phospholipids, β-alanine, and N-acetylaspartic acid—against prior findings, our framework not only confirms previously reported HER2-positive signatures but also highlights platform-consistent patterns and novel combinations of metabolites, providing actionable insights for precision diagnostics (page 14).

**General Changes Implemented Across the Manuscript**

In addition to point-specific revisions, several broader updates were made to improve clarity, consistency, and readability of the manuscript:

- **Removal of Pre-trained Model Section:** We removed references to “pre-trained” models, since after employing nested repeated stratified cross-validation and reporting 95% confidence intervals, performance was excellent, making this step unnecessary and potentially confusing for readers.
- **Feature Visualization Adjustment:** The “Top 15” metabolite features were reduced to Top 10 for better visualization and interpretability in summary figures.
- **Workflow Presentation:** The workflows for Concatenated, Ensemble, and Deep Forest strategies were merged into one multi-panel figure, improving clarity and cohesion.
- **Supplementary Material Restructuring:** As suggested by Reviewer #1, most detailed tables have been moved to the Supporting Information, while only key results remain in the main text.
- **Expanded Methods Section:** Additional details were added on:
  - Metabolomics multimodal datasets (origin, class balance, sample selection)
  - Data curation, missing value handling, and normalization
  - Model validation strategies and overfitting prevention measures (including dropout, early stopping, L1/L2 regularization, and permutation ROC tests)
- **Pathway Integration:** KEGG pathway analysis related to **HER2-positive breast cancer** was incorporated, along with a schematic illustration of HER2-positive expression, which aligned with the metabolites identified in our study.
