# Supplementary material for: Unlocking precision diagnostics: A multimodal framework integrating metabolomics with advanced machine learning techniques
Source: PLoS One. 2026 Jun 15;21(6):e0318473. doi: 10.1371/journal.pone.0318473 (PMC13268153; doi:10.1371/journal.pone.0318473)
Supplement: Attachment — Submitted filename: Minor Revision Comments.docx [file pone.0318473.s012.docx]

**Minor Revision Comments**

1. **Terminology consistency**
   Please ensure consistent use of terminology throughout the manuscript, particularly with respect to Deep Transfer Learning. In a few places, related terms such as “pre-trained model” and “transfer learning” appear interchangeably, which may lead to minor ambiguity for readers.
2. **Conciseness of narrative sections**
   Although the manuscript has improved substantially, parts of the Introduction and Discussion remain overly detailed. Minor condensation would improve readability and narrative flow without compromising scientific clarity.
3. **Pathway-level integration visibility**
   The inclusion of pathway schematics in the Supplementary Information strengthens biological interpretation. A brief reference to these figures in the main text would help guide readers and improve accessibility.
4. **Clarification of translational scope**
   The Discussion could be further strengthened by adding one or two sentences outlining practical considerations for translational application, such as analytical scalability, cost, and feasibility of implementing multimodal metabolomics workflows in clinical or diagnostic settings.
5. **Supplementary material refinement**
   Please recheck the Supplementary Material for minor formatting inconsistencies, typographical errors, and uniformity in figure/table labeling to ensure consistency with the main manuscript.

**Final Recommendation**

The authors have satisfactorily addressed the major concerns raised in the previous review. The manuscript is scientifically sound, methodologically rigorous, and significantly improved. I recommend acceptance after minor revisions.
