# Supplementary material for: Unlocking precision diagnostics: A multimodal framework integrating metabolomics with advanced machine learning techniques
Source: PLoS One. 2026 Jun 15;21(6):e0318473. doi: 10.1371/journal.pone.0318473 (PMC13268153; doi:10.1371/journal.pone.0318473)
Supplement: Peer review history

# Peer review: Decision Letter 0

**DOI:** 10.1371/journal.pone.0318473.r001

**Authors:** Ch Ratnasekhar

28 Aug 2025

PONE-D-25-02568

Unlocking Precision Diagnostics: A Multimodal Framework Integrating Metabolomics with Advanced Machine Learning Techniques

PLOS ONE

Dear Dr. Salek,

Thank you for submitting your manuscript to PLOS ONE. After careful consideration, we feel that it has merit but does not fully meet PLOS ONE’s publication criteria as it currently stands. Therefore, we invite you to submit a revised version of the manuscript that addresses the points raised during the review process.

Please submit your revised manuscript by Oct 12 2025 11:59PM. If you will need more time than this to complete your revisions, please reply to this message or contact the journal office at plosone@plos.org. When you're ready to submit your revision, log on to https://www.editorialmanager.com/pone/ and select the 'Submissions Needing Revision' folder to locate your manuscript file.

Please include the following items when submitting your revised manuscript:

- A rebuttal letter that responds to each point raised by the academic editor and reviewer(s). You should upload this letter as a separate file labeled 'Response to Reviewers'.
- A marked-up copy of your manuscript that highlights changes made to the original version. You should upload this as a separate file labeled 'Revised Manuscript with Track Changes'.
- An unmarked version of your revised paper without tracked changes. You should upload this as a separate file labeled 'Manuscript'.

If you would like to make changes to your financial disclosure, please include your updated statement in your cover letter. Guidelines for resubmitting your figure files are available below the reviewer comments at the end of this letter.

If applicable, we recommend that you deposit your laboratory protocols in protocols.io to enhance the reproducibility of your results. Protocols.io assigns your protocol its own identifier (DOI) so that it can be cited independently in the future. For instructions see: https://journals.plos.org/plosone/s/submission-guidelines#loc-laboratory-protocols. Additionally, PLOS ONE offers an option for publishing peer-reviewed Lab Protocol articles, which describe protocols hosted on protocols.io. Read more information on sharing protocols at https://plos.org/protocols?utm_medium=editorial-email&utm_source=authorletters&utm_campaign=protocols.

We look forward to receiving your revised manuscript by implementing the reviewers suggestions.

Kind regards,

Ch Ratnasekhar, Ph.D.

Academic Editor

PLOS ONE

Journal Requirements:

When submitting your revision, we need you to address these additional requirements.

1.Please ensure that your manuscript meets PLOS ONE's style requirements, including those for file naming. The PLOS ONE style templates can be found at

https://journals.plos.org/plosone/s/file?id=wjVg/PLOSOne_formatting_sample_main_body.pdf and

https://journals.plos.org/plosone/s/file?id=ba62/PLOSOne_formatting_sample_title_authors_affiliations.pdf

2. Thank you for uploading your study's underlying data set. Unfortunately, the repository you have noted in your Data Availability statement does not qualify as an acceptable data repository according to PLOS's standards.

At this time, please upload the minimal data set necessary to replicate your study's findings to a stable, public repository (such as figshare or Dryad) and provide us with the relevant URLs, DOIs, or accession numbers that may be used to access these data. For a list of recommended repositories and additional information on PLOS standards for data deposition, please see https://journals.plos.org/plosone/s/recommended-repositories.

3. When completing the data availability statement of the submission form, you indicated that you will make your data available on acceptance. We strongly recommend all authors decide on a data sharing plan before acceptance, as the process can be lengthy and hold up publication timelines. Please note that, though access restrictions are acceptable now, your entire data will need to be made freely accessible if your manuscript is accepted for publication. This policy applies to all data except where public deposition would breach compliance with the protocol approved by your research ethics board. If you are unable to adhere to our open data policy, please kindly revise your statement to explain your reasoning and we will seek the editor's input on an exemption. Please be assured that, once you have provided your new statement, the assessment of your exemption will not hold up the peer review process.

4. Please include captions for your Supporting Information files at the end of your manuscript, and update any in-text citations to match accordingly. Please see our Supporting Information guidelines for more information: http://journals.plos.org/plosone/s/supporting-information.

5. If the reviewer comments include a recommendation to cite specific previously published works, please review and evaluate these publications to determine whether they are relevant and should be cited. There is no requirement to cite these works unless the editor has indicated otherwise.

Additional Editor Comments:

Reviewer #1:

Unlocking Precision Diagnostics: A Multimodal Framework Integrating Metabolomics with Advanced Machine Learning Techniques

This manuscript explores integration methodologies of multimodal metabolomics data (LC-MS, GC-MS, NMR) using advanced machine learning (ML) strategies, including Deep Transfer Learning (DTL), Multiple Kernel Learning (MKL), Deep Forest, and ensemble methods. It aims to identify significant biomarkers for HER2-positive breast cancer.

Major Revisions

1. Add external validation cohort

Issue: The study uses only internal cross-validation (nested repeated stratified CV). No independent cohort was used for validation.

• Validate the final models (especially MKL and pre-trained ANN) on an external metabolomics dataset, preferably breast cancer samples with HER2 classification.

• If external data is unavailable, apply leave-one-group-out cross-validation or nested LOOCV as a surrogate.

• Clarify in the methods if any form of data leakage was avoided when concatenating features and reusing datasets.

2. Address overfitting and model complexity

Issue: Exceptionally high metrics (AUC > 0.99, Sensitivity = 1) across all models raise overfitting concerns, especially with a sample size of 253.

• Provide learning curves with standard deviation bars across folds.

• Report confidence intervals (e.g., 95% CI) for all performance metrics.

• Include feature distribution plots to show class separation (e.g., PCA or UMAP of encoded features).

3. Comparative analysis of biomarkers

Issue: The metabolite panel derived using SHAP is not evaluated as a biomarker classifier.

• Perform multivariate ROC analysis using the top 6 statistically significant metabolites (e.g., PC 30:1, PE 32:1).

• Report AUC, sensitivity, specificity, PPV, and NPV of this biomarker panel on a held-out test set.

4. Include pathway and network enrichment

Issue: Metabolites are individually discussed but not contextualized in a systems biology framework.

• Use KEGG Mapper, Reactome, or MetaboAnalyst to map significant metabolites to metabolic pathways.

• Include an enrichment plot or network visualization showing upregulated/downregulated pathways in HER2+.

5. Clarify sample source and preprocessing

Issue: The sample origin (METAcancer FP7) is not well described.

• Clearly define the sample types (e.g., tumor tissue or serum), number per class (HER2+, HER2−), and selection criteria.

• Provide platform-specific preprocessing steps, especially normalization, batch correction, and imputation.

6. Simplify figures and results presentation

Issue: Many figures (especially SHAP and violin plots) are complex and crowded.

• Combine SHAP bar plots and violin plots into a single summary figure per platform.

• Move multi-metric tables to Supplementary and summarize top results in the main text.

7. Improve biological interpretation and literature support

Issue: Some claims are speculative or unsupported.

• Avoid speculative claims unless supported by in vivo or in vitro literature (e.g., role of β-alanine in HER2+ tumors).

• Cite relevant mechanistic studies to support observed metabolite changes (e.g., role of SCD1, gluconic acid in HER2 biology).

Suggested experimental validation (For Future or Revision Inclusion)

If the authors have access to lab facilities or collaborators, the following experimental validations can be proposed:

1. Targeted metabolite quantification

• Use LC-MS/MS MRM to quantitatively validate levels of 3–6 key metabolites (e.g., PC 30:1, β-alanine, MTA) in an independent breast cancer cohort.

2. Immunohistochemistry / western blot

• Evaluate SCD1 expression or related enzymes (e.g., FASN, ACC1) in HER2+ vs HER2− breast tumors using IHC or western blot to support lipidomic findings.

3. Cell line validation

• Measure metabolite changes in HER2+ vs HER2− breast cancer cell lines (e.g., SKBR3 vs MCF7) using targeted metabolomics.

4. Functional assays

• Test the effect of β-alanine or MTA supplementation on HER2+ cell viability, oxidative stress, or apoptosis to validate their proposed roles.

8. Integrate a multi-omics comparison or benchmarking

Issue: The study presents multimodal integration but does not benchmark it against other omics layers (e.g., transcriptomics or proteomics).

• Discuss how the metabolomics-based integration compares to existing multi-omics models used in HER2 breast cancer classification.

• Include benchmarking with publicly available multi-omics data (like from TCGA-BRCA) if possible, or discuss limitations.

9. Enhance model interpretability with biological networks

Issue: SHAP values provide feature importance but not biological context.

• Create a network diagram linking top metabolites to pathways, enzymes, and relevant genes using tools like Cytoscape or MetScape.

• Highlight connections between lipid metabolism, amino acid metabolism, and HER2 signaling.

10. Incorporate confidence intervals and statistical testing

Issue: Performance metrics lack statistical rigor.

• Include 95% confidence intervals (CI) for all reported metrics (Accuracy, F1, AUC, etc.).

• Apply DeLong’s test for comparing ROC curves between models.

• Perform statistical comparison of models (e.g., t-test or ANOVA) to prove DTL and MKL significantly outperform baseline models.

11. Justify sample size and power

Issue: Only 253 samples used; sample size justification is weak.

• Perform a priori power analysis or cite effect size calculations to justify that the sample size is sufficient.

• Use learning curves to show performance saturation i.e., adding more samples doesn’t significantly improve results.

12. Analyze model robustness to noise

Issue: No robustness analysis is presented.

• Perform a noise injection experiment by randomly permuting a subset of features or labels.

• Report how robust each model is to such perturbations. This shows real-world generalizability.

13. Address batch effects and instrument drift

Issue: Integrating three metabolomics platforms assumes no batch effects.

• Describe steps taken for batch effect correction, alignment, and normalization across LC-MS, GC-MS, and NMR data.

• If possible, run PCA before and after correction to show improved integration.

14. Improve supplementary material quality

Issue: Supplementary figures and tables are disorganized or minimal.

• Provide complete data matrices (anonymized), model parameters, confusion matrices, and full SHAP value lists in supplementary files.

• Include a GitHub or CodeOcean repository with full code, model weights, and a Jupyter Notebook for reproducibility.

15. Add clinical relevance and utility section

Issue: The manuscript lacks practical discussion on translational application.

• Add a section that discusses how the model could be translated into a diagnostic workflow.

• Include limitations such as cost, scalability, and time required to run a multimodal ML pipeline in a clinical lab.

16. Conduct subgroup or stratified analysis

Issue: The HER2 group may contain heterogeneous clinical subtypes.

• Perform stratified model performance evaluation based on menopausal status, tumor grade, or ER/PR status (if available).

• This will improve personalization and support precision diagnostics claims.

17. Explain the role of each ML algorithm choice

Issue: Many ML models are applied, but not all are justified.

• Provide a table comparing models, explaining why each was chosen, its strengths, and what it adds.

• Remove any redundant methods if they don’t add unique value (e.g., if Deep Forest and Ensemble give nearly identical outputs).

18. Improve the discussion of limitations

Issue: Current limitations are only briefly acknowledged.

• Explicitly discuss limitations like class imbalance, lack of histopathological validation, retrospective data, and multi-center variability.

• A transparent discussion builds credibility.

Reviewer #2:

I have read, with interest, the article, the title of which is Unlocking Precision Diagnostics: A Multimodal Framework That Integrates Metabolomics with Novel Machine Learning Methods. Although the study indicates that the Deep Transfer Learning (DTL) and Multiple Kernel Learning (MKL) methods were carried out comprehensively to integrate metabolomics data sets of LC-MS, GC-MS, and NMR platforms, I would like to make some comments regarding its novelty claims.

Combining several omics modalities, especially in the context of cancer biomarker research, is well established in the literature. Similarly, DTL and MKL have been practically used in cancer detection, as well as other biomedical data analysis tasks. Earlier metabolomics research has also identified phospholipids (phosphatidylcholine, phosphatidylethanolamine, sphingomyelin) and amino acid related metabolites ( 2 -alanine, N-acetylaspartic acid, etc.) as possible markers in HER2-positive individuals.

Moreover, inherent differences in sensitivity and metabolite coverage of the three analytical platforms are not critically discussed in the study. LC-MS/MS specifically, has been noted to be significantly more sensitive than GC-MS or NMR, often identifying more metabolites at lower abundance. Unless their differences during integration are appropriately weighted or the effect on biomarker discovery than the comparison of modalities in the integrated model, it may be biased, with LC-MS/MS taking most of the signal.

I would urge the authors to better define and justify their methodological developments beyond the application of extant algorithms, to directly mention the sources of bias due to the variation in the sensitivity of the analytical platforms, and to contextualize their biomarker observations relative to earlier results to underscore what is actually new. It will assist the readers and reviewers in better understanding the meaning of their framework for precision diagnostics.

Additional Editor Comments:

I recommend authors to implement comments suggested by reviewers and highlight how their work contribute to the advancement in this field in the introduction part, and resubmit the manuscript for further consideration by addressing this decision letter in their cover letter during their new submission. If authors can not implement suggestions, provide justification/explanation why it can not be done.

[Note: HTML markup is below. Please do not edit.]

Reviewer's Responses to Questions

**Comments to the Author**

1. Is the manuscript technically sound, and do the data support the conclusions?

The manuscript must describe a technically sound piece of scientific research with data that supports the conclusions. Experiments must have been conducted rigorously, with appropriate controls, replication, and sample sizes. The conclusions must be drawn appropriately based on the data presented.

Reviewer #1: Partly

Reviewer #2: No

**********

2. Has the statistical analysis been performed appropriately and rigorously?

Reviewer #1: No

Reviewer #2: No

**********

3. Have the authors made all data underlying the findings in their manuscript fully available?

The PLOS Data policy requires authors to make all data underlying the findings described in their manuscript fully available without restriction, with rare exception (please refer to the Data Availability Statement in the manuscript PDF file). The data should be provided as part of the manuscript or its supporting information, or deposited to a public repository. For example, in addition to summary statistics, the data points behind means, medians and variance measures should be available. If there are restrictions on publicly sharing data—e.g. participant privacy or use of data from a third party—those must be specified.

Reviewer #1: No

Reviewer #2: Yes

**********

4. Is the manuscript presented in an intelligible fashion and written in standard English?

PLOS ONE does not copyedit accepted manuscripts, so the language in submitted articles must be clear, correct, and unambiguous. Any typographical or grammatical errors should be corrected at revision, so please note any specific errors here.

Reviewer #1: No

Reviewer #2: Yes

**********

5. Review Comments to the Author

Please use the space provided to explain your answers to the questions above. You may also include additional comments for the author, including concerns about dual publication, research ethics, or publication ethics. (Please upload your review as an attachment if it exceeds 20,000 characters)

Reviewer #1: Unlocking Precision Diagnostics: A Multimodal Framework Integrating Metabolomics with Advanced Machine Learning Techniques

This manuscript explores integration methodologies of multimodal metabolomics data (LC-MS, GC-MS, NMR) using advanced machine learning (ML) strategies, including Deep Transfer Learning (DTL), Multiple Kernel Learning (MKL), Deep Forest, and ensemble methods. It aims to identify significant biomarkers for HER2-positive breast cancer.

Major Revisions

1. Add external validation cohort

Issue: The study uses only internal cross-validation (nested repeated stratified CV). No independent cohort was used for validation.

Required Revision:

• Validate the final models (especially MKL and pre-trained ANN) on an external metabolomics dataset, preferably breast cancer samples with HER2 classification.

• If external data is unavailable, apply leave-one-group-out cross-validation or nested LOOCV as a surrogate.

• Clarify in the methods if any form of data leakage was avoided when concatenating features and reusing datasets.

2. Address overfitting and model complexity

Issue: Exceptionally high metrics (AUC > 0.99, Sensitivity = 1) across all models raise overfitting concerns, especially with a sample size of 253.

Required Revision:

• Provide learning curves with standard deviation bars across folds.

• Report confidence intervals (e.g., 95% CI) for all performance metrics.

• Include feature distribution plots to show class separation (e.g., PCA or UMAP of encoded features).

3. Comparative analysis of biomarkers

Issue: The metabolite panel derived using SHAP is not evaluated as a biomarker classifier.

required revision:

• Perform multivariate ROC analysis using the top 6 statistically significant metabolites (e.g., PC 30:1, PE 32:1).

• Report AUC, sensitivity, specificity, PPV, and NPV of this biomarker panel on a held-out test set.

4. Include pathway and network enrichment

Issue: Metabolites are individually discussed but not contextualized in a systems biology framework.

Required Revision:

• Use KEGG Mapper, Reactome, or MetaboAnalyst to map significant metabolites to metabolic pathways.

• Include an enrichment plot or network visualization showing upregulated/downregulated pathways in HER2+.

5. Clarify sample source and preprocessing

Issue: The sample origin (METAcancer FP7) is not well described.

Required Revision:

• Clearly define the sample types (e.g., tumor tissue or serum), number per class (HER2+, HER2−), and selection criteria.

• Provide platform-specific preprocessing steps, especially normalization, batch correction, and imputation.

6. Simplify figures and results presentation

Issue: Many figures (especially SHAP and violin plots) are complex and crowded.

Required Revision:

• Combine SHAP bar plots and violin plots into a single summary figure per platform.

• Move multi-metric tables to Supplementary and summarize top results in the main text.

7. Improve biological interpretation and literature support

Issue: Some claims are speculative or unsupported.

Required Revision:

• Avoid speculative claims unless supported by in vivo or in vitro literature (e.g., role of β-alanine in HER2+ tumors).

• Cite relevant mechanistic studies to support observed metabolite changes (e.g., role of SCD1, gluconic acid in HER2 biology).

Suggested experimental validation (For Future or Revision Inclusion)

If the authors have access to lab facilities or collaborators, the following experimental validations can be proposed:

1. Targeted metabolite quantification

• Use LC-MS/MS MRM to quantitatively validate levels of 3–6 key metabolites (e.g., PC 30:1, β-alanine, MTA) in an independent breast cancer cohort.

2. Immunohistochemistry / western blot

• Evaluate SCD1 expression or related enzymes (e.g., FASN, ACC1) in HER2+ vs HER2− breast tumors using IHC or western blot to support lipidomic findings.

3. Cell line validation

• Measure metabolite changes in HER2+ vs HER2− breast cancer cell lines (e.g., SKBR3 vs MCF7) using targeted metabolomics.

4. Functional assays

• Test the effect of β-alanine or MTA supplementation on HER2+ cell viability, oxidative stress, or apoptosis to validate their proposed roles.

8. Integrate a multi-omics comparison or benchmarking

Issue: The study presents multimodal integration but does not benchmark it against other omics layers (e.g., transcriptomics or proteomics).

Revision:

• Discuss how the metabolomics-based integration compares to existing multi-omics models used in HER2 breast cancer classification.

• Include benchmarking with publicly available multi-omics data (like from TCGA-BRCA) if possible, or discuss limitations.

9. Enhance model interpretability with biological networks

Issue: SHAP values provide feature importance but not biological context.

Revision:

• Create a network diagram linking top metabolites to pathways, enzymes, and relevant genes using tools like Cytoscape or MetScape.

• Highlight connections between lipid metabolism, amino acid metabolism, and HER2 signaling.

10. Incorporate confidence intervals and statistical testing

Issue: Performance metrics lack statistical rigor.

Revision:

• Include 95% confidence intervals (CI) for all reported metrics (Accuracy, F1, AUC, etc.).

• Apply DeLong’s test for comparing ROC curves between models.

• Perform statistical comparison of models (e.g., t-test or ANOVA) to prove DTL and MKL significantly outperform baseline models.

11. Justify sample size and power

Issue: Only 253 samples used; sample size justification is weak.

Revision:

• Perform a priori power analysis or cite effect size calculations to justify that the sample size is sufficient.

• Use learning curves to show performance saturation i.e., adding more samples doesn’t significantly improve results.

12. Analyze model robustness to noise

Issue: No robustness analysis is presented.

Revision:

• Perform a noise injection experiment by randomly permuting a subset of features or labels.

• Report how robust each model is to such perturbations. This shows real-world generalizability.

13. Address batch effects and instrument drift

Issue: Integrating three metabolomics platforms assumes no batch effects.

Revision:

• Describe steps taken for batch effect correction, alignment, and normalization across LC-MS, GC-MS, and NMR data.

• If possible, run PCA before and after correction to show improved integration.

14. Improve supplementary material quality

Issue: Supplementary figures and tables are disorganized or minimal.

Revision:

• Provide complete data matrices (anonymized), model parameters, confusion matrices, and full SHAP value lists in supplementary files.

• Include a GitHub or CodeOcean repository with full code, model weights, and a Jupyter Notebook for reproducibility.

15. Add clinical relevance and utility section

Issue: The manuscript lacks practical discussion on translational application.

Revision:

• Add a section that discusses how the model could be translated into a diagnostic workflow.

• Include limitations such as cost, scalability, and time required to run a multimodal ML pipeline in a clinical lab.

16. Conduct subgroup or stratified analysis

Issue: The HER2 group may contain heterogeneous clinical subtypes.

Revision:

• Perform stratified model performance evaluation based on menopausal status, tumor grade, or ER/PR status (if available).

• This will improve personalization and support precision diagnostics claims.

17. Explain the role of each ML algorithm choice

Issue: Many ML models are applied, but not all are justified.

Revision:

• Provide a table comparing models, explaining why each was chosen, its strengths, and what it adds.

• Remove any redundant methods if they don’t add unique value (e.g., if Deep Forest and Ensemble give nearly identical outputs).

18. Improve the discussion of limitations

Issue: Current limitations are only briefly acknowledged.

Revision:

• Explicitly discuss limitations like class imbalance, lack of histopathological validation, retrospective data, and multi-center variability.

• A transparent discussion builds credibility.

Reviewer #2: I have read, with interest, the article, the title of which is Unlocking Precision Diagnostics: A Multimodal Framework That Integrates Metabolomics with Novel Machine Learning Methods. Although the study indicates that the Deep Transfer Learning (DTL) and Multiple Kernel Learning (MKL) methods were carried out comprehensively to integrate metabolomics data sets of LC-MS, GC-MS, and NMR platforms, I would like to make some comments regarding its novelty claims.

Combining several omics modalities, especially in the context of cancer biomarker research, is well established in the literature. Similarly, DTL and MKL have been practically used in cancer detection, as well as other biomedical data analysis tasks. Earlier metabolomics research has also identified phospholipids (phosphatidylcholine, phosphatidylethanolamine, sphingomyelin) and amino acid related metabolites ( 2 -alanine, N-acetylaspartic acid, etc.) as possible markers in HER2-positive individuals.

Moreover, inherent differences in sensitivity and metabolite coverage of the three analytical platforms are not critically discussed in the study. LC-MS/MS specifically, has been noted to be significantly more sensitive than GC-MS or NMR, often identifying more metabolites at lower abundance. Unless their differences during integration are appropriately weighted or the effect on biomarker discovery than the comparison of modalities in the integrated model, it may be biased, with LC-MS/MS taking most of the signal.

I would urge the authors to better define and justify their methodological developments beyond the application of extant algorithms, to directly mention the sources of bias due to the variation in the sensitivity of the analytical platforms, and to contextualize their biomarker observations relative to earlier results to underscore what is actually new. It will assist the readers and reviewers in better understanding the meaning of their framework for precision diagnostics.

**********

6. PLOS authors have the option to publish the peer review history of their article (what does this mean?). If published, this will include your full peer review and any attached files.

If you choose “no”, your identity will remain anonymous but your review may still be made public.

**Do you want your identity to be public for this peer review?**  For information about this choice, including consent withdrawal, please see our Privacy Policy.

Reviewer #1: No

Reviewer #2: No

**********

[NOTE: If reviewer comments were submitted as an attachment file, they will be attached to this email and accessible via the submission site. Please log into your account, locate the manuscript record, and check for the action link "View Attachments". If this link does not appear, there are no attachment files.]

While revising your submission, please upload your figure files to the Preflight Analysis and Conversion Engine (PACE) digital diagnostic tool, https://pacev2.apexcovantage.com/. PACE helps ensure that figures meet PLOS requirements. To use PACE, you must first register as a user. Registration is free. Then, login and navigate to the UPLOAD tab, where you will find detailed instructions on how to use the tool. If you encounter any issues or have any questions when using PACE, please email PLOS at figures@plos.org. Please note that Supporting Information files do not need this step.

Attachment Submitted filename: plosOne comments.docx

# Author comment: Author response to Decision Letter 1

**DOI:** 10.1371/journal.pone.0318473.r002

5 Dec 2025

detailed repoposes are in the file: Reply to reviewersNov25.docx and below

Dear Editor-in-Chief and the reviewers,

We sincerely thank the reviewers for their insightful and constructive feedback. We carefully revised the manuscript and implemented changes according to the reviewers' requests wherever possible, while providing reasoned responses in cases where certain suggestions could not be applied directly. To facilitate transparency, we highlighted the revisions within the manuscript:

• Green highlights indicate changes made in response to Reviewer #1.

• Yellow highlights indicate changes made in response to Reviewer #2.

Below, we provide point-by-point responses to each comment.

Reviewer #1

1- The study only uses internal cross-validation (nested repeated stratified CV). Could the authors validate the final models (especially MKL and pre-trained ANN) on an independent external metabolomics cohort, ideally including breast cancer samples with HER2 classification? If external data are unavailable, could alternative strategies, such as leave-one-group-out CV or nested LOOCV be applied? Additionally, please clarify how data leakage was prevented when combining features from multiple platforms and reusing datasets.

Response:

We thank the reviewer for this constructive suggestion, which underscores the importance of robust validation in multimodal machine learning studies. We fully agree that external validation on an independent HER2-annotated cohort with matched LC-MS, GC-MS, and NMR platforms would be ideal; however, despite an extensive search of public repositories (e.g., Metabolomics Workbench, GNPS, TCGA, etc.), no suitable dataset was available. This limitation is common in specialized metabolomics, where the availability of such multi-platform cohort datasets remains scarce.

To address this, we applied repeated stratified cross-validation and nested stratified cross-validation (depending on the model), which provide statistically stable estimates of generalization while balancing class distributions. Compared with leave-one-out CV (LOOCV), these approaches are both more computationally feasible in multimodal integration (avoiding repeated kernel re-computation or full retraining per sample) and less prone to high-variance estimates in moderate datasets (n ≈ 423 post-oversampling). In preliminary tests, LOOCV yielded unstable and inconsistent metrics, whereas stratified CV produced robust and reproducible performance (e.g., test AUC > 0.99 for HER2 classification).

Finally, to prevent data leakage, all preprocessing steps (e.g., normalization, feature selection, autoencoder dimensionality reduction) were performed strictly within training folds of the outer CV loop, with test data held out until final evaluation. Platform features were integrated only after per-fold processing, ensuring complete separation between training and test sets.

2-The exceptionally high metrics reported (AUC > 0.99, Sensitivity = 1) across all models raise concerns about potential overfitting, particularly given the original sample size of 253. Could you provide:

- Learning curves with standard deviation bars across folds?

- Confidence intervals (e.g., 95% CI) for all performance metrics?

- Feature distribution plots (e.g., PCA or UMAP of encoded features) to illustrate class separation?

Response:

We appreciate the reviewer's valuable feedback. Below, we detail the changes implemented to address the reviewer's requests and improve the clarity of our methods, including the additional analysis requested.

High AUC values: For models showing near-perfect AUC (~1), we performed permutation ROC p-value testing, and in all cases, the p-values were effectively 0. This indicates that the observed performance is statistically significant and not attributable to chance.

Sensitivity: The apparent sensitivity = 1 observed for HER2 status reflects the dataset composition, where the majority of samples are HER2-negative, resulting in no false negatives.

Confidence intervals and learning curves: We have updated all model metrics to include 95% confidence intervals, calculated via bootstrapping or across folds, to provide a robust measure of uncertainty and generalization. In addition, learning curves with standard deviation bars across folds are now included for MKL and Deep Transfer Learning models, demonstrating stable convergence without signs of overfitting.

Dataset details and oversampling: After applying oversampling to balance minority classes, the total sample size increased to 423, which improves model training stability and subgroup representation (e.g., HER2-positive, ER-negative). We have updated the Methods section with more detailed information on dataset composition and created a summary table to better illustrate class distributions.

Feature distribution plots: We note that PCA and UMAP are unsupervised dimensionality reduction methods and, for complex multi-platform metabolomics data, are best suited for exploratory visualization rather than for validating class separation. Accordingly, we did not rely on these plots to support model performance. Instead, we ensured robust generalization through multiple methodological safeguards, including:

- Data normalization and batch normalization to mitigate platform-specific variation and stabilize training.

- Dropout layers to prevent co-adaptation of neurons and reduce overfitting.

- Hyperparameter tuning via repeated stratified cross-validation, including optimization of network units and dropout rates.

- L1 and L2 regularization to penalize large weights and improve generalization.

- Early stopping to halt training once validation performance plateaued, avoiding overfitting.

- Permutation ROC tests confirming that high AUC values represent the true predictive signal. Together, these measures confirm that the observed high performance—such as sensitivity of 1 for HER2 status —arises from the underlying data structure and rigorous model design, not overfitting or methodological artifacts.

3-Comparative analysis of biomarkers

Issue: The metabolite panel derived using SHAP is not evaluated as a biomarker classifier.

- Perform multivariate ROC analysis using the top 6 statistically significant metabolites (e.g., PC 30:1, PE 32:1).

- Report AUC, sensitivity, specificity, PPV, and NPV of this biomarker panel on a held-out test set.

Response:

We appreciate the reviewer’s suggestion to evaluate the top SHAP-ranked metabolites as a standalone biomarker panel. In our study, the workflow focused on extracting informative latent features using an autoencoder bottleneck, then applying SHAP analysis to identify the top 30 contributing metabolites across the three platforms, instead of restricting the analysis to a predefined small set of metabolites.

Additionally, in our previously published work in Oct 2024, using the LC-MS lipidomics dataset, we performed ROC and MCC analyses at the individual metabolite level, together with effect size and fold-change assessments under defined p-value thresholds, published here (doi.org/10.1038/s41598-024-71439-7). In the present study, beyond SHAP ranking, we further refined the feature set using statistical filtering (effect size and p-value < 0.05), highlighting six key metabolites. These results are now added, and summarized in a forest plot (Fig. 5, Page 23), which provides a clear visualization of both effect size and statistical significance for these metabolites (previously presented in tabular form).

- 4-Include pathway and network enrichment

Issue: Metabolites are individually discussed but not contextualized in a systems biology framework.

- Use KEGG Mapper, Reactome, or MetaboAnalyst to map significant metabolites to metabolic pathways.

- Include an enrichment plot or network visualization showing upregulated/downregulated pathways in HER2+.

Response:

We agree with the reviewer that contextualizing metabolite-level findings within a systems biology framework is essential, and we’ve added a new figure to reflect this. Our study primarily focused on employing advanced machine learning methods to integrate multi-platform metabolomics data and pinpoint metabolites of translational significance. The reliability of the selected metabolites was supported through comparison with previous studies, rather than relying solely on enrichment and visualization. Nonetheless, in response to the reviewer’s suggestion, we have now included pathway-level information in the Supporting Information. Specifically, we illustrate the HER2 signalling pathway to show how activation of key proteins and enzymes may relate to the observed metabolic alterations. We also schematically present the involvement of monounsaturated phospholipids (e.g., PC 30:1) in HER2-driven SCD1 upregulation, alongside changes in glutamate and β-alanine pathways in HER2-positive cases. These representations complement our machine learning framework and offer mechanistic insights, without being the main basis for metabolite selection (S3 Fig Page 9).

5-Clarify sample source and preprocessing

Issue: The sample origin (METAcancer FP7) is not well described.

- Clearly define the sample types (e.g., tumor tissue or serum), number per class (HER2+, HER2−), and selection criteria.

- Provide platform-specific preprocessing steps, especially normalization, batch correction, and imputation.

Response:

We thank the reviewer for their attention to this point. We have now expanded the Methods section to provide a clearer description of the sample origin (METAcancer FP7), including the sample types (e.g., tumor tissue or serum), the number of samples per class (HER2-positive, HER2-negative), and other selection criteria as requested (page 7). Additionally, we have summarized the dataset composition, including raw and oversampled samples, in a new table for clarity (S1 Table Page 1). Platform-specific preprocessing steps are now also described in greater detail, covering normalization, batch correction, and imputation procedures, to ensure transparency of the analysis (page 8).

6- Simplify figures and results presentation

Issue: Many figures (especially SHAP and violin plots) are complex and crowded.

- Combine SHAP bar plots and violin plots into a single summary figure per platform.

- Move multi-metric tables to Supplementary and summarize top results in the main text.

Response:

We thank the reviewer for this suggestion to consolidate the figures. We have now streamlined the figures and results presentation as follows:

- For each platform, we now provide a single summary figure that highlights the top 10 SHAP-ranked metabolites (reduced from the original 15) along with their corresponding violin plots, illustrating both contribution and class-wise distribution (Fig. 4, p. 22).

- Comprehensive multi-metric evaluation tables have been moved to the Supplementary Material, while only the key results are summarized in the main text. Metrics for the ANN-based deep learning model are retained in the main manuscript (Table 1, p. 25).

- The workflows for Concatenation, Ensemble, and Deep Forest approaches have been consolidated into a single schematic (Fig. 1, p. 16).

7- Improve biological interpretation and literature support

Issue: Some claims are speculative or unsupported.

- Avoid speculative claims unless supported by in vivo or in vitro literature (e.g., role of β-alanine in HER2+ tumors).Cite relevant mechanistic studies to support observed metabolite changes (e.g., role of SCD1, gluconic acid in HER2 biology).

Suggested experimental validation (For Future or Revision Inclusion)

Response:

We thank the reviewer for stressing the importance of grounding results in biological interpretations from cited literature. In response to the comment, we have made the following change and added additional resources, see new changes/citations highlighted in yellow in the main text:

- Expanded literature support: We have revised the Discussion to include additional mechanistic and clinical studies supporting the roles of the selected key metabolites (e.g., PC 30:1, β-alanine, MTA) in HER2 biology, reinforcing their biological and translational relevance.

- Targeted metabolite focuses and future plan: We focus on the key metabolites identified by our workflow and propose targeted metabolite analysis as a future direction for precision medicine and translational research. This includes potential validation in independent cohorts or clinical settings to confirm their utility as biomarkers and to guide therapeutic strategies.

8- Integrate a multi-omics comparison or benchmarking

Issue: The study presents multimodal integration but does not benchmark it against other omics layers (e.g., transcriptomics or proteomics).

• Discuss how the metabolomics-based integration compares to existing multi-omics models used in HER2 breast cancer classification.

• Include benchmarking with publicly available multi-omics data (like from TCGA-BRCA) if possible, or discuss limitations.

Response:

Although this is a great suggestion, carrying out such tasks is beyond our scope and the available data. While integration with other omics layers (e.g., transcriptomics or proteomics) could further enhance biological insight and benchmarking potential, such integration requires either matched samples across platforms or advanced strategies, such as multi-head autoencoders with shared latent embeddings. These approaches allow weighting and mapping across heterogeneous datasets, analogous to our current multi-platform metabolomics workflow (Page 24).

In the present study, we focused on a single omics type with relatively lower heterogeneity compared to multi-omics datasets. Harmonizing data across multiple omics, particularly when drawn from different sources, remains a key challenge for generalization. We have now highlighted the implementation of multi-omics integration as a future direction in the Discussion section, emphasizing its potential for expanding our framework in precision medicine and translational analysis.

9. Enhance model interpretability with biological networks

Issue: SHAP values provide feature importance but not biological context.

- Create a network diagram linking top metabolites to pathways, enzymes, and relevant genes using tools like Cytoscape or MetScape.

Response:

As described earlier, we have now added a new schematic illustration of the pathways related to the selected metabolites, highlighting connections to key enzymes and relevant biological processes (S3 Fig, Page 9).

10. Incorporate confidence intervals and statistical testing

Issue: Performance metrics lack statistical rigor.

• Include 95% confidence intervals (CI) for all reported metrics (Accuracy, F1, AUC, etc.).

• Apply DeLong’s test for comparing ROC curves between models.

• Perform statistical comparison of models (e.g., t-test or ANOVA) to prove DTL and MKL significantly outperform baseline models.

Response:

We share the same passion and believe in rigor for informatics, analytical, and statistical-based results, and we take further suggestions on board. In our revised analysis, we have included 95% confidence intervals (CI) for all reported performance metrics (Accuracy, F1, AUC, etc.), calculated using bootstrap resampling across folds, which provides a robust estimate of metric variability and model generalization.

For ROC evaluation, we applied permutation-based p-value testing rather than DeLong’s test. Permutation testing is particularly suitable in our setting because it does not rely on distributional assumptions and directly assesses the significance of observed AUC values under the null hypothesis of no predictive signal. In all cases, the permutation p-values were effectively 0, confirming that the high AUCs reflect true predictive performance.

Regarding statistical comparisons between models, conventional tests such as t-tests or ANOVA are not appropriate here, because repeated cross-validation metrics are not independent and may violate test assumptions. Therefore, we focused on reporting confidence intervals and permutation-based significance as more reliable and interpretable measures of model performance.

11. Justify sample size and power

Issue: Only 253 samples used; sample size justification is weak.

Attachment Submitted filename: Reply to reviewersNov25.docx

# Peer review: Decision Letter 1

**DOI:** 10.1371/journal.pone.0318473.r003

**Authors:** Kwang-Sig Lee

7 Apr 2026

<p>Unlocking Precision Diagnostics: A Multimodal Framework Integrating Metabolomics with Advanced Machine Learning Techniques

PONE-D-25-02568R1

Dear Dr. Salek,

We’re pleased to inform you that your manuscript has been judged scientifically suitable for publication and will be formally accepted for publication once it meets all outstanding technical requirements.

Within one week, you’ll receive an e-mail detailing the required amendments. When these have been addressed, you’ll receive a formal acceptance letter and your manuscript will be scheduled for publication.

An invoice will be generated when your article is formally accepted. Please note, if your institution has a publishing partnership with PLOS and your article meets the relevant criteria, all or part of your publication costs will be covered. Please make sure your user information is up-to-date by logging into Editorial Manager at Editorial Manager® and clicking the ‘Update My Information' link at the top of the page. For questions related to billing, please contact billing support.

If your institution or institutions have a press office, please notify them about your upcoming paper to help maximize its impact. If they’ll be preparing press materials, please inform our press team as soon as possible -- no later than 48 hours after receiving the formal acceptance. Your manuscript will remain under strict press embargo until 2 pm Eastern Time on the date of publication. For more information, please contact onepress@plos.org.

Kind regards,

Kwang-Sig Lee

Academic Editor

PLOS One

Additional Editor Comments (optional):

Reviewers' comments:

Reviewer's Responses to Questions

**Comments to the Author**

1. If the authors have adequately addressed your comments raised in a previous round of review and you feel that this manuscript is now acceptable for publication, you may indicate that here to bypass the “Comments to the Author” section, enter your conflict of interest statement in the “Confidential to Editor” section, and submit your "Accept" recommendation.

Reviewer #1: All comments have been addressed

**********

2. Is the manuscript technically sound, and do the data support the conclusions?

The manuscript must describe a technically sound piece of scientific research with data that supports the conclusions. Experiments must have been conducted rigorously, with appropriate controls, replication, and sample sizes. The conclusions must be drawn appropriately based on the data presented.

Reviewer #1: Yes

**********

3. Has the statistical analysis been performed appropriately and rigorously?

Reviewer #1: Yes

**********

4. Have the authors made all data underlying the findings in their manuscript fully available?

The PLOS Data policy requires authors to make all data underlying the findings described in their manuscript fully available without restriction, with rare exception (please refer to the Data Availability Statement in the manuscript PDF file). The data should be provided as part of the manuscript or its supporting information, or deposited to a public repository. For example, in addition to summary statistics, the data points behind means, medians and variance measures should be available. If there are restrictions on publicly sharing data—e.g. participant privacy or use of data from a third party—those must be specified.

Reviewer #1: Yes

**********

5. Is the manuscript presented in an intelligible fashion and written in standard English?

PLOS ONE does not copyedit accepted manuscripts, so the language in submitted articles must be clear, correct, and unambiguous. Any typographical or grammatical errors should be corrected at revision, so please note any specific errors here.

Reviewer #1: Yes

**********

6. Review Comments to the Author

Please use the space provided to explain your answers to the questions above. You may also include additional comments for the author, including concerns about dual publication, research ethics, or publication ethics. (Please upload your review as an attachment if it exceeds 20,000 characters)

Reviewer #1: Minor Revision Comments

1. Terminology consistency

Please ensure consistent use of terminology throughout the manuscript, particularly with respect to deep transfer learning. In a few places, related terms such as “pre-trained model” and “transfer learning” appear interchangeably, which may lead to minor ambiguity for readers.

2. Conciseness of narrative sections

Although the manuscript has improved substantially, parts of the introduction and discussion remain overly detailed. Minor condensation would improve readability and narrative flow without compromising scientific clarity.

3. Pathway-level integration visibility

The inclusion of pathway schematics in the supplementary information strengthens biological interpretation. A brief reference to these figures in the main text would help guide readers and improve accessibility.

4. Clarification of translational scope

The discussion could be further strengthened by adding one or two sentences outlining practical considerations for translational application, such as analytical scalability, cost, and feasibility of implementing multimodal metabolomics workflows in clinical or diagnostic settings.

5. Supplementary material refinement

Please recheck the Supplementary Material for minor formatting inconsistencies, typographical errors, and uniformity in figure/table labeling to ensure consistency with the main manuscript.

**********

7. PLOS authors have the option to publish the peer review history of their article (what does this mean?). If published, this will include your full peer review and any attached files.

If you choose “no”, your identity will remain anonymous but your review may still be made public.

**Do you want your identity to be public for this peer review?**  For information about this choice, including consent withdrawal, please see our Privacy Policy.

Reviewer #1: No

**********

Attachment Submitted filename: Minor Revision Comments.docx

# Editor report: Acceptance letter

**DOI:** 10.1371/journal.pone.0318473.r004

**Authors:** Kwang-Sig Lee

PONE-D-25-02568R1

PLOS One

Dear Dr. Salek,

I'm pleased to inform you that your manuscript has been deemed suitable for publication in PLOS One. Congratulations! Your manuscript is now being handed over to our production team.

At this stage, our production department will prepare your paper for publication. This includes ensuring the following:

* All references, tables, and figures are properly cited

* All relevant supporting information is included in the manuscript submission,

* There are no issues that prevent the paper from being properly typeset

You will receive further instructions from the production team, including instructions on how to review your proof when it is ready. Please keep in mind that we are working through a large volume of accepted articles, so please give us a few days to review your paper and let you know the next and final steps.

Lastly, if your institution or institutions have a press office, please let them know about your upcoming paper now to help maximize its impact. If they'll be preparing press materials, please inform our press team within the next 48 hours. Your manuscript will remain under strict press embargo until 2 pm Eastern Time on the date of publication. For more information, please contact onepress@plos.org.

You will receive an invoice from PLOS for your publication fee after your manuscript has reached the completed accept phase. If you receive an email requesting payment before acceptance or for any other service, this may be a phishing scheme. Learn how to identify phishing emails and protect your accounts at https://explore.plos.org/phishing.

If we can help with anything else, please email us at customercare@plos.org.

Thank you for submitting your work to PLOS ONE and supporting open access.

Kind regards,

PLOS ONE Editorial Office Staff

on behalf of

Professor Kwang-Sig Lee

Academic Editor

PLOS One
